# A junctophilin-caveolin interaction enables efficient coupling between ryanodine receptors and BK_Ca_ channels in the Ca^2+^ microdomain of vascular smooth muscle

**DOI:** 10.1074/jbc.RA119.008342

**Published:** 2019-07-15

**Authors:** Takanori Saeki, Yoshiaki Suzuki, Hisao Yamamura, Hiroshi Takeshima, Yuji Imaizumi

**Affiliations:** ‡ Department of Molecular and Cellular Pharmacology, Graduate School of Pharmaceutical Sciences, Nagoya City University, Nagoya 467-8603, Japan; § Department of Biological Chemistry, Graduate School of Pharmaceutical Sciences, Kyoto University, Kyoto 606-8501, Japan

**Keywords:** vascular smooth muscle cells, calcium channel, potassium channel, calcium imaging, molecular imaging, single-molecule biophysics, patch clamp, fluorescence resonance energy transfer (FRET), caveolin, blood pressure

## Abstract

Functional coupling between large-conductance Ca^2+^-activated K^+^ (BK_Ca_) channels in the plasma membrane (PM) and ryanodine receptors (RyRs) in the sarcoplasmic reticulum (SR) is an essential mechanism for regulating mechanical force in most smooth muscle (SM) tissues. Spontaneous Ca^2+^ release through RyRs (Ca^2+^ sparks) and subsequent BK_Ca_ channel activation occur within the PM-SR junctional sites. We report here that a molecular interaction of caveolin-1 (Cav1), a caveola-forming protein, with junctophilin-2 (JP2), a bridging protein between PM and SR, positions BK_Ca_ channels near RyRs in SM cells (SMCs) and thereby contributes to the formation of a molecular complex essential for Ca^2+^ microdomain function. Approximately half of all Ca^2+^ sparks occurred within a close distance (<400 nm) from fluorescently labeled JP2 or Cav1 particles, when they were moderately expressed in primary SMCs from mouse mesenteric artery. The removal of caveolae by genetic *Cav1* ablation or methyl-β-cyclodextrin treatments significantly reduced coupling efficiency between Ca^2+^ sparks and BK_Ca_ channel activity in SMCs, an effect also observed after JP2 knockdown in SMCs. A 20-amino acid-long region in JP2 appeared to be essential for the observed JP2-Cav1 interaction, and we also observed an interaction between JP2 and the BK_Ca_ channel. It can be concluded that the JP2-Cav1 interaction provides a structural and functional basis for the Ca^2+^ microdomain at PM-SR junctions and mediates cross-talk between RyRs and BK_Ca_ channels, converts local Ca^2+^ sparks into membrane hyperpolarization, and contributes to stabilizing resting tone in SMCs.

## Introduction

Ca^2+^ microdomains refer various types of transient elevation of intracellular Ca^2+^ concentration ([Ca^2+^]*_i_*) and are a key element of Ca^2+^ signaling (1). Junctophilins (JPs)^2^ are a family of structural proteins that span the immediate subcellular gaps between the plasma membrane (PM) and endo/sarcoplasmic reticulum (ER/SR) (2, 3). The JP1 and JP2 isoforms have obligatory roles in the junctional membrane complexes (JMCs) formed under the transverse- (T-) tubular system in striated muscle cells. They enable skeletal and cardiac myocytes to translate conformational changes in voltage-gated Ca^2+^ channels (VDCCs) in PM and Ca^2+^ influx through VDCC, respectively, to a marked elevation as a Ca^2+^ microdomain via highly effective Ca^2+^ release from SR through ryanodine receptors (RyR) during excitation-contraction (E-C) coupling (4–6). JP3 and JP4 are strongly expressed in neurons and also important for the regulation of cellular Ca^2+^ signaling (7).

In the N termini of all JPs, eight conserved motifs, each consisting of 14 amino acid residues, have been named “membrane occupation and recognition nexus” (MORN) motifs. These motifs in the N termini selectively bind to T-tubular membranes and C-terminal transmembrane segments anchor the opposite end to the ER/SR membrane (2). Among the four subtypes of JPs (JP1–4), JP2 is predominantly expressed in cardiac myocytes (8). Its function is essential for physiological E-C coupling (2), and it is altered in progressive hypertrophic cardiomyopathy (9, 10).

JP2 mRNA expression has been reported in some smooth muscle (SM) tissues (2). However, in contrast to striated muscles, SM cells (SMCs) lack the T-tubular system, and, thus, the role of JP2 currently remains unknown. We previously suggested that caveolae, Ω-shaped structures on PM, serve as a structural component responsible for functions of Ca^2+^ microdomains, and may play significant roles in the control of Ca^2+^ signaling, excitability, and contractility by facilitating the functional coupling of ion channel complexes in vascular SMCs (VSMCs) (11, 12). For example, localized and spontaneous Ca^2+^ release from RyR, referred to as Ca^2+^ sparks, activates large-conductance Ca^2+^-activated K^+^ (BK_Ca_) channels in caveolae. As a result, spontaneous transient outward currents (STOCs) are generated to induce membrane hyperpolarization (13, 14), leading to the suppression of VDCC activity and stabilization of resting tone in SM tissues (15). Caveolae are implicated in structurally confining action potential Ca^2+^ signals and may enable efficient, bidirectional Ca^2+^-mediated cross-talk between the cell surface and SR (11, 16).

Caveolin isoforms (Cav1, 2, and 3) are differentially expressed in tissue-dependent manners and are essential for the stability and function of caveolae (17). Cav3, which is the isoform that is predominantly expressed in skeletal and cardiac myocytes, forms caveolae in the extra T-tubular PM. In contrast, Cav1 may be the key molecule for composing caveolae in SMCs based on observations that a genetic deficiency in Cav1 results in the abolishment of caveolae (18) and that Cav1 accumulates various types of signal molecules in caveolae (17). In SMCs, a JMC-like structure has been detected in specific parts of SR elements located just beneath caveolae by EM analyses (19).

Based on previous findings (2, 12, 20), we hypothesized that JP2 in SMCs is involved in the formation of distinct molecular complex responsible for Ca^2+^ microdomain function within JMCs. Key molecules, such as VDCC, Cav1, BK_Ca_ channels, and RyR, may be included in the functional molecular complex in JMCs. Here, fluorescent imaging based on total internal reflection fluorescence (TIRF) microscopy and whole-cell patch clamp recordings reveal that JP2 functions as a critical structural component enabling efficient localized translation of Ca^2+^ sparks to STOCs, thus effectively regulating resting tone in VSMCs.

## Results

### JP2 binds to Cav1 in a caveolar structure-dependent manner

**Figure 1.**
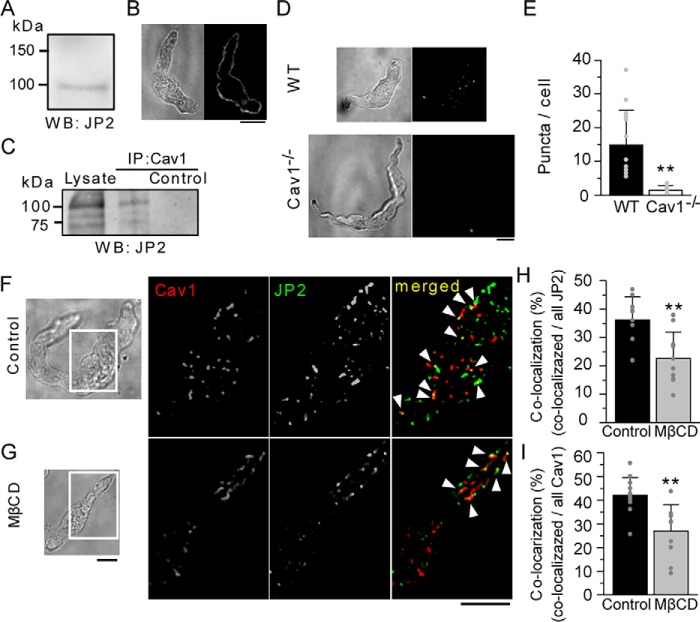
**Abundant expression of JP2 in MASMCs, and its interaction with Cav1.** *A,* a Western blot analysis using a protein lysate isolated from the mouse mesenteric artery. *B,* an immunofluorescent image of a freshly isolated mMASMC obtained using a confocal microscope and its transmitted light image. *C,* co-IP indicating molecular coupling between JP2 and Cav1. Extracts of the rat mesenteric artery were processed for IP with anti-Cav1 antibodies. Bound proteins were solubilized and analyzed on SDS-PAGE, followed by immunoblotting for JP2. *D,* epifluorescent images obtained by PLA experiments for the interaction between JP2 and Cav1 in MASMCs from WT (*top*) and Cav1^−/−^ mice (*bottom*). *E,* the number of puncta per cell was summarized (WT: *n* = 14, Cav1^−/−^: *n* = 10). *F* and *G,* JP2 and Cav1 in freshly isolated mMASMCs were labeled with specific antibodies, and then visualized using a TIRF microscope. Caveolae were disrupted by the treatment with 10 mm MβCD (*G*). Fluorescent signals corresponding to JP2, Cav1, and their co-localization are colored in *green*, *red*, and *yellow* (denoted by *arrowheads*), respectively. *H* and *I,* ratios of the number of co-localizing particles to those of all JP2 (*H*) or Cav1 (*I*) particles in control and MβCD-treated myocytes. **, *p* < 0.01; the Student's *t* test. *Scale bars* indicate 10 μm (*B*, *D*, *F*, and *G*).

JP2 is abundantly expressed in striated muscles such as cardiac muscles (2). On the other hand, the expression of JP2 has only been reported at the mRNA level in the SM of the stomach and lung (2). Therefore, our initial experiments were performed to examine the mRNA expression of JP2 in the mouse mesenteric artery. The mRNA expression of JP2 in the mouse mesenteric artery was abundantly detected, in contrast to that of JP1 (Fig. S1*A*). Western blotting (Fig. 1*A*) and immunocytochemical staining (Fig. 1*B*) showed the abundant protein expression of JP2 and its prominent distribution along PM in mouse mesenteric artery SMCs (mMASMCs).

Based on our quantitative PCR analyses, the major Cav isoform expressed in mMASMCs was Cav1 (Fig. S1*B*), as has been reported in airway SM (21). Because a part of the caveola is located very close to SR in SMCs (19), we investigated whether JP2 binds to Cav1. Co-immunoprecipitation (co-IP) assays revealed an interaction between JP2 and Cav1 in rat mesenteric artery SM tissues (Fig. 1*C*). Moreover, the application of *in situ* proximity ligation assays (PLAs, see “Experimental procedures”) for JP2 and Cav1 in mMASMCs resulted in the appearance of abundant green fluorescent puncta in epifluorescent images, indicating co-localization of these two molecules within 40 nm (Fig. 1*D*). The number of puncta in MASMCs from WT mice was significantly larger than in Cav1^−/−^ (WT: 14.8 ± 10.3 puncta/cell, *n* = 14, Cav1^−/−^: 1.5 ± 1.4 puncta/cell, *n* = 10, *p* < 0.01, Fig. 1*E*). These results suggest that JP2 and Cav1 closely interact each other in native myocytes.

Single molecular imaging using a TIRF microscope was performed to visualize the precise distribution of JP2 and Cav1. These two proteins in freshly isolated mMASMCs were each stained with a specific antibody in SMCs. TIRF images visualized fluorescent particles existing on PM (Fig. 1*F*). Some of the fluorescent particles of JP2 co-localized with those of Cav1 in mMASMCs (yellow particles indicated by *arrowheads* in Fig. 1*F*). To investigate this interaction in more detail, the effects of 10 mm methyl-β-cyclodextrin (MβCD), which removes cholesterol from PM and disrupts caveolae, were examined (Fig. 1*G*). The ratio of co-localizing particles was significantly reduced by the MβCD treatment (the ratio of co-localization against all JP2 particles: control, 36.2 ± 8.1%, *n* = 12; MβCD, 22.6 ± 9.3%, *n* = 10, *p* < 0.01; the ratio of co-localization against all Cav1 particles, control, 42.1 ± 7.4%, *n* = 10; MβCD, 27.0 ± 11.1%, *n* = 12, *p* < 0.01; Fig. 1, *H* and *I*).

**Figure 2.**
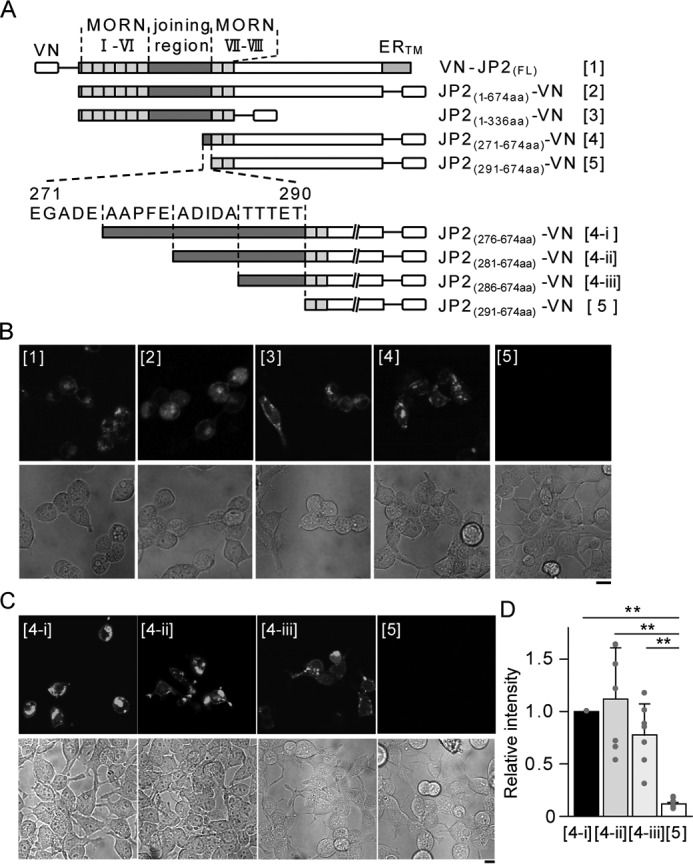
**Essential binding sites of JP2 for the interaction with Cav1.** *A,* the JP2 truncation constructs ([*1*] to [*5*]) used in the present study are shown. MORN motifs, the joining regions, and ER transmembrane domains (ER_TM_) are indicated. *B* and *C,* a series of representative confocal images of the BiFC assay. Fluorescent signals in the *top panels* indicate the complementation of VN and VC. Corresponding transmitted light images are shown in the *bottom*. Representative images from three independent experiments are shown. *D,* the fluorescence intensity of complimented Venus within regions of interest (*ROI*) in each image was divided by that of the Hoechst signal. The Venus/Hoechst ratio of the mutants ([*4-ii*], [*4-iii*], and [*5*]) was normalized to that of JP2(276–674aa)-VN ([*4-i*]). Seven sets of images ([*4-i*], [*4-ii*], [*4-iii*], and [*5*]) were acquired from three independent experiments in epifluorescent fields. *, *p* < 0.05; **, *p* < 0.01; one-way ANOVA followed by Tukey's test. *Scale bars* indicate 10 μm (*B* and *C*).

The specific region of JP2 responsible for the interaction with Cav1 was examined by bimolecular fluorescent complementation (BiFC) analyses (22). VN and VC are the N- and C-terminal halves, respectively, of the Venus fluorescent protein. The N terminus of full-length JP2 (VN-JP2) or the C termini of a series of truncated JP2 constructs (JP2_(X-Y)_-VN) were labeled with VN (Fig. 2*A*, *upper*). The N terminus of Cav1 (VC-Cav1) was labeled with VC. These constructs were co-expressed in HEK293 cells and visualized by confocal microscopy. The consistent detection of fluorescent signals from the reconstituted Venus protein by VN-VC coupling strongly suggested a molecular interaction between JP2 or truncated JP2 and Cav1. The deletion of 271–290 residues within the joining region (compare [*4*] and [*5*] in Fig. 2*A*) removed molecular coupling between JP2 and Cav1, as shown in Fig. 2*B*. Further molecular analyses using deletion mutants (mutants [*4-i*], [*4-ii*], [*4-iii*], [*5*] in Fig. 2, *A*, *lower*, and *C*) revealed that ^286^TTTET^290^ was important for JP2-Cav1 binding (*n* = 7, mutant [*5*] *versus* [*4-i*], *p* < 0.01; mutant [*5*] *versus* [*4-ii*], [*4-iii*] *p* < 0.05; Fig. 2*D*). Collectively, these results strongly suggest that JP2 (^286^TTTET^290^) interacts with Cav1 in caveolae in mMASMCs.

### Ca^2+^ spark-generating sites are proximal to JMCs in mMASMCs.

**Figure 3.**
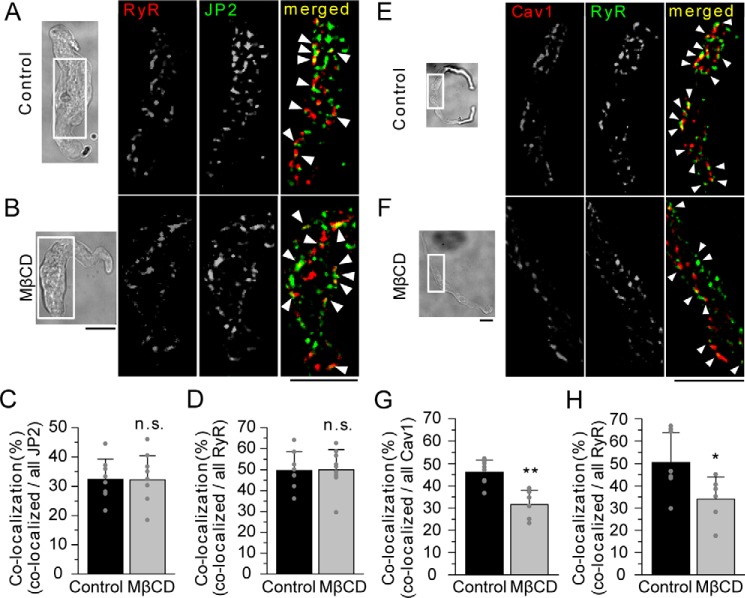
**TIRF imaging of the co-localization of RyR-JP2 and Cav1-RyR in mMASMCs.** *A* and *B,* RyR and JP2 in freshly isolated mMASMCs were identified by immunostaining using antibodies specific to each protein and a TIRF microscope. Cells were nontreated or treated with MβCD before staining. TIRF images obtained from the area surrounded by the *white line* in transmitted light images (*left*) are shown. Fluorescent signals corresponding to RyR, JP2, and their co-localization are colored in *red*, *green*, and *yellow* (denoted by *arrowheads*), respectively. *C* and *D,* the ratio of the number of co-localizing particles to that of all JP2 (*C*) or RyR (*D*) particles in control (*n* = 8) and MβCD-treated myocytes (*n* = 9); *p* > 0.05; *t* test. *E* and *F,* RyR and Cav1 in freshly isolated mMASMCs were labeled with each specific antibody and visualized using a TIRF microscope. Fluorescent signals corresponding to RyR, Cav1, and their co-localization are shown in *green*, *red*, and *yellow* (denoted by *arrowheads*), respectively. *G* and *H,* the ratio of the number of co-localizing particles to that of all Cav1 (*G*) or RyR (*H*) particles in control and MβCD myocytes. *, *p* < 0.05; **, *p* < 0.01; *t* test. *Scale bars* indicate 10 μm (*A*, *B*, *E*, and *F*).

The next series of experiments focused on the relationship between JP2 and RyR (Fig. 3), which has been reported in striated muscles (23). In freshly isolated mMASMCs, the fluorescent particles of RyR were abundantly detected in TIRF images, indicating very close localization to PM (in the evanescent wave area within 200 nm of the bottom of the chamber; Fig. 3, *A* and *B*) (24). The application of MβCD had no effect on this co-localization (Fig. 3, *B–D*) and the number of fluorescent particles of RyR and JP2 (Table S1), suggesting that JP2 binds to RyR in a caveolar-independent manner. The possibility of the co-localization of Cav1 with RyR was also examined in mMASMCs (Fig. 3, *E* and *F*). The ratio of co-localizing particles was significantly lower in MβCD-pretreated myocytes than in the control (for Cav1, control, 46.2 ± 5.4%, *n* = 8; MβCD, 31.6 ± 6.5%, *n* = 6, *p* < 0.01; for RyR, control, 50.5 ± 13.3%; MβCD, 34.1 ± 9.9%, *p* < 0.05; Fig. 3, *G* and *H*). These results suggest that the JP2-mediated co-localization of Cav1 and RyR depends on the integrity of the caveolar structure.

**Figure 4.**
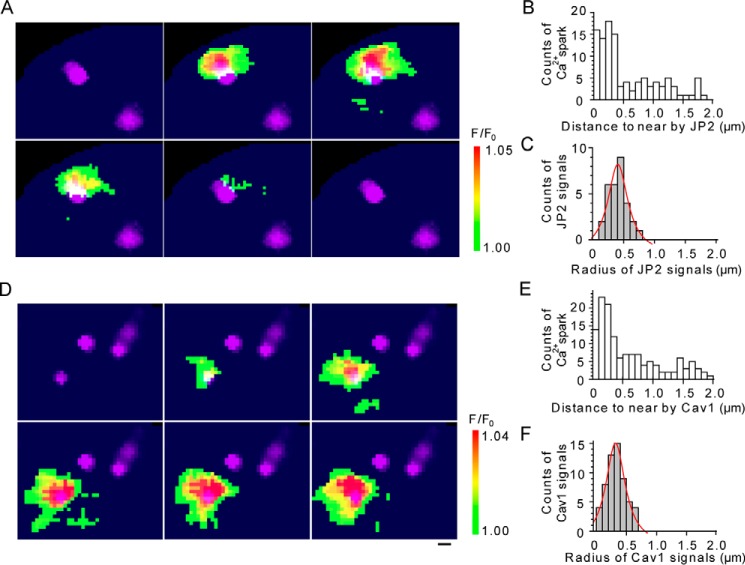
**TIRF images of Ca^2+^ sparks and those of JP2 or Cav1 in mMASMCs.** *A* and *D,* Ca^2+^ sparks in 40 mm [K^+^]*_o_* were detected with fluo-4/AM using a TIRF microscope. Continuous TIRF images of Ca^2+^ sparks were obtained at 27.2-ms intervals. Ca^2+^-spark images were merged with those of mCherry-JP2 or mCherry-cav1 (*purple*), which were separately recorded in the same cells. *B* and *E,* the distance from the center of a Ca^2+^-spark site to the center of the nearest mCherry-JP2 or mCherry-cav1 particles were measured. The number of observed sparks *versus* the distance is demonstrated in the distribution histogram (108 sparks from 12 mCherry-JP2-expressing cells, cav1; 146 sparks from 15 mCherry-cav1-expressing cells). *C* and *F,* the radii of mCherry-JP2 or mCherry-cav1 particles located closest to Ca^2+^-spark sites were measured (30 mCherry-JP2 particles, 59 mCherry-cav1 particles). The distribution histogram of radii is demonstrated and fit by the Lorenz function (*red curves*). *Scale bars* indicate 500 nm (*A* and *D*).

To gain functional insights into this coupling, Ca^2+^ dynamics and the location of JP2 or Cav1 were simultaneously recorded in a mMASMC using TIRF microscopy. To prevent the excessive expression of mCherry-tagged JP2 or Cav1 in mMASMCs, the amount of cDNA for transfection was carefully adjusted as reported previously (25). Our goal was to reveal the spatiotemporal relationship between Ca^2+^-spark sites and JMCs or caveolae. The Ca^2+^ sparks were blocked by 100 μm tetracaine, a RyR inhibitor, but not by 3 μm xestospongin C, an IP_3_ receptor inhibitor (*n* = 3 for each, not shown in figures). JP2 or Cav1 labeled with mCherry was transiently expressed in primary-cultured mMASMCs, and Ca^2+^ sparks were recorded by fluo-4 imaging (Fig. 4*A* and Movie S1). Based on the kinetic analysis, the Ca^2+^ signals are identified as Ca^2+^ sparks (time to peak: 62.0 ± 18.1 ms in mCherry-JP2 expressed cells, 51.8 ± 14.3 ms mCherry-Cav1-expressing cells; full width at half-maximum (FWHM): 2.4 ± 0.9 μm in mCherry-JP2-expressing cells, 2.3 ± 0.8 μm in mCherry-Cav1–expressing cells). These parameters were very similar to previous findings (time to peak: 20–95 ms (26), FWHM: 2–4 μm (27)).

Two-dimensional distances from spark-generating sites detected as the Ca^2+^ spark-initiating point in each movie to the fluorescent peak of the closest mCherry-JP2 particles were measured and summarized in Fig. 4*B*. We noted that the mean radius of mCherry-JP2 particles was 455 nm (*r*^2^ = 0.92, Lorenz fit, Fig. 4*C*). A total of 60.2% of Ca^2+^ sparks (65/108 sparks from 12 cells) overlapped with mCherry-JP2 particles. Moreover, 61.1% of Ca^2+^ spark-generating sites (22/36 spark-generating sites from 12 cells) were located within 460 nm of the peak (usually the center) points of mCherry-JP2 signals. Similarly, 47.3% of Ca^2+^ sparks (69/146 sparks from 15 cells, Fig. 4*E*) overlapped with the fluorescent particles of mCherry-Cav1 in mMASMCs expressing mCherry-Cav1 (Fig. 4*D* and Movie S2). The mean radius of mCherry-Cav1 was 368 nm (Fig. 4*F*). Based on analyses of spark-generating sites, 35.8% of the sites (24/67 from 15 cells) were located within 370 nm of the peak point (typically the center) of mCherry-Cav1 signals. The density of Ca^2+^ spark-generating sites (sites/μm^2^) in the JP2 signals (JP2in) and outside of them (JP2out) was 0.276 ± 0.257 and 0.007 ± 0.007, respectively (*p* < 0.01). The density in the Cav1 signals (Cav1in) and the outside (Cav1out) was 0.517 ± 0.454 and 0.019 ± 0.010, respectively (*p* < 0.01) (Table S2). These results indicate that a large proportion of Ca^2+^ spark-generating sites, which may include RyR clusters on SR, closely localized with caveolae (<368 nm) and JP2 signals (<455 nm) in JMC.

### The JP2-BK_Ca_ channel complex is assembled by Cav1 in caveolae

BK_Ca_ channels are preferentially localized in caveolae by the interaction with Cav1 (12, 28) and are efficiently activated by Ca^2+^ sparks arising from RyR in SR (12, 29). Our assumption is that JP2 is the key molecule functionally linking BK_Ca_ channels with RyR in the Ca^2+^ microdomain.

Thus, to examine the exact relationship between JP2 and the BK_Ca_ channel, we performed co-IP assays, which clearly demonstrated a molecular interaction between JP2 and BK_Ca_ channels in the rat mesenteric artery (Fig. S2*A*) and in HEK293 cells expressing GFP-JP2 and BK_Ca_ channels (Fig. S2*B*). Our BiFC analyses based on VN-tagged JP2 and the VC-tagged α subunit of BK_Ca_ channels (BKα) revealed reconstructed Venus fluorescence along the PM in HEK293 cells, in which VN-JP2 and BKα-VC were both expressed (Fig. S2*C*). The results using truncated mutants of VN-JP2 revealed a novel molecular interaction of JP2 joining region with BKα-VC, as well as Cav1-VC in Fig. 1, in living cells (Fig. S2, *D* and *E*).

**Figure 5.**
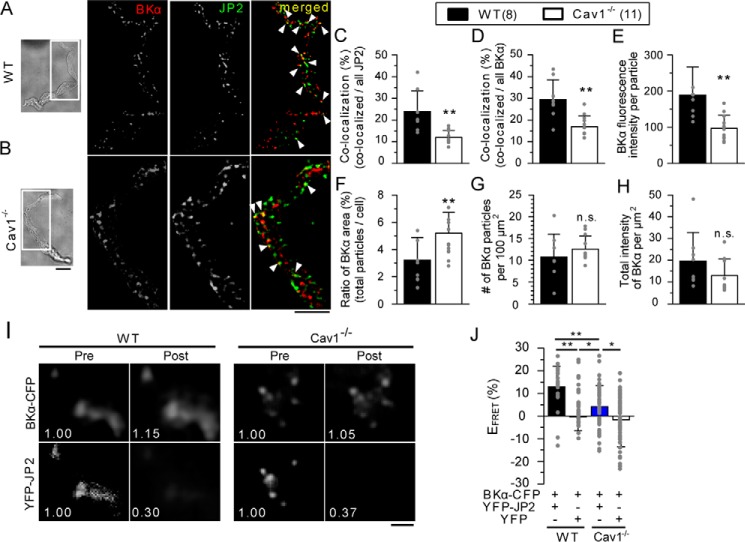
**The JP2 protein and BK_Ca_ channels co-assembled in caveolae in mMASMCs.** *A* and *B,* JP2 and BKα subunits in freshly isolated mMASMCs from WT (*A*) or Cav1^−/−^ (*B*) mice were labeled with specific antibodies, and then visualized using a TIRF microscope. Fluorescent signals corresponding to BK_Ca_, JP2, and their co-localization are colored in *red*, *green*, and *yellow* (denoted by *arrowheads*), respectively. *C* and *D,* ratios of the number of co-localizing particles to those of all JP2 (*C*) or BKα (*D*) particles in WT and Cav1^−/−^ myocytes. *E–H,* the averaged fluorescence intensity in particles of BKα (*E*), the percentage of the integrated area occupied by BKα particles *versus* the cell area in TIRF images (*F*), the number of BKα particles per unit area (100 μm^2^) in TIRF images (*G*), and the integrated intensity of BKα particles per unit area (*H*) are shown. The number of cells examined was 8 for WT and 11 for Cav1^−/−^ (*C–H*). **, *p* < 0.01; the Student's *t* test. *I,* a FRET analysis was performed in mMASMCs co-expressing YFP-JP2 and BKα-CFP from WT and Cav1^−/−^. Panels show YFP-JP2 and BKα-CFP emissions before (*Pre*) and after (*Post*) selective YFP photobleaching. The numerical value in each image indicates fluorescence intensity relative to that before bleaching. *J,* summarized data of E_FRET_ in WT and Cav1^−/−^ (YFP-JP2 + BKα-CFP in WT myocytes: 37 particles, 5 cells; YFP + BKα-CFP in WT myocytes: 51 particles, 11 cells, YFP-JP2 + BKα-CFP in Cav1^−/−^ myocytes: 64 particles, 7 cells; YFP + BKα-CFP: 25 particles, 5 cells in Cav1^−/−^ myocytes). *, *p* < 0.05; **, *p* < 0.01; two-way ANOVA was applied to the analysis (see Table S3). The effect of Cav1 on the FRET values (Effect 1) were compared in WT and Cav^−/−^ was measured as the molecular interaction between YFP-JP2 and BKα-CFP. The effect of YFP on the molecular interaction between BKα-CFP and YFP-JP2 (Effect 2) was also compared in WT and Cav1^−/−^. The interaction between Effect 1 and Effect 2 was statistically significant (*F*-test, *p* < 0.05). Thus, the statistical significance between the four groups was examined by Tukey's test. *Scale bars* indicate 10 μm (*A* and *B*) and 2 μm (*I*), respectively. *n.s*., not significant.

We then investigated whether Cav1/caveola contributed to the molecular complex formation of the BK_Ca_ channel with JP2. Immunostaining analyses with TIRF microscopy showed that BK_Ca_ channels are colocalized with JP2 in freshly isolated mMASMCs (Fig. 5*A*). We then investigated whether Cav1/caveolae contributed to the molecular complex formation of BK_Ca_ channels with JP2 using Cav1 knockout mice (Cav1^−/−^). The co-localization of JP2 and BKα was also detected in Cav1^−/−^ myocytes (Fig. 5*B*). The ratio of their co-localization *versus* all BKα particles was significantly decreased by the Cav1 deficiency (WT, 29.4 ± 3.2%, *n* = 8; Cav1^−/−^, 16.7 ± 1.5%, *n* = 11, *p* < 0.01 *versus* WT), although the number of fluorescent particles of JP2 and BKα were not altered (Table S1). The ratio of co-localizing particles *versus* all JP2 particles was also decreased by the Cav1 deficiency (WT, 23.9 ± 9.6%, *n* = 8; Cav1^−/−^, 12.0 ± 3.3%, *n* = 11, *p* < 0.01 *versus* control; Fig. 5, *C* and *D*). Similar results were obtained when caveolae were disrupted by the MβCD pretreatment (Fig. S3).

The average fluorescent intensity per single BKα particle in Cav1^−/−^ (97.2 ± 36.1, *n* = 11) was significantly lower than that in WT (189.0 ± 77.6, *n* = 8, *p* < 0.01) (Fig. 5*E*). The percentage of the integrated area occupied by BKα particles *versus* the total TIRF image area was also calculated and compared between WT and Cav1^−/−^ (Fig. 5*F*). The percentage of BKα in WT (3.2 ± 1.7%, *n* = 8) was significantly smaller than that in Cav1^−/−^ (5.2 ± 1.5%, *n* = 11, *p* < 0.01). In contrast, the number of BKα particles per unit area (100 μm^2^) of TIRF images and the integrated intensity of BKα particles per unit area were not significantly different between WT and Cav1^−/−^ (number of particles: 10.7 ± 5.3 in WT, *n* = 8, and 12.6 ± 3.0 in Cav1^−/−^, *n* = 11, *p* > 0.05; intensity: 19.6 ± 13.1 in WT, *n* = 8, and 12.9 ± 7.6 in Cav1^−/−^, *n* = 11, *p* > 0.05; Fig. 5, *G* and *H*). These results indicate that BK_Ca_ channels are more highly concentrated within local areas (detected as single particles in this experiment) in WT than in Cav1^−/−^. Therefore, Cav1/caveolae are important for the accumulation of BK_Ca_ channels and form a structural basis to facilitate Ca^2+^ microdomain function by promoting BK_Ca_-JP2 coupling. Moreover, FRET analyses using TIRF microscopy were performed based on acceptor photobleaching methods (Fig. 5, *I* and *J*) (25). In WT myocytes co-expressing YFP-JP2 + BKα-CFP, the fluorescence intensity of BKα-CFP was significantly increased after the bleaching of YFP-JP2 (FRET efficiency (E_FRET_) in WT myocytes: YFP-JP2 + BKα-CFP, 12.9 ± 9.1%, 37 particles in 5 cells; YFP + BKα-CFP as a negative control, −0.34 ± 6.19%, 51 particles in 11 cells, *p* < 0.01). This increase in BKα-CFP fluorescence intensity after bleaching was significantly attenuated in Cav1^−/−^ myocytes (E_FRET_ in Cav1^−/−^ myocytes, YFP-JP2 + BKα-CFP, 4.2 ± 9.1%, 64 particles in 7 cells, *p* < 0.01 *versus* WT myocytes; YFP + BKα-CFP, −1.6 ± 12.0%, 25 particles in 5 cells, *p* < 0.05 *versus* YFP-JP2 + BKα-CFP in Cav1^−/−^ myocytes). These results indicate that Cav1 facilitates the selective localization of BK_Ca_ channels in caveolae and enhances the formation of a molecular complex with JP2 in mMASMCs.

### JP2 mediates molecular interactions between Cav1 and RyR

**Figure 6.**
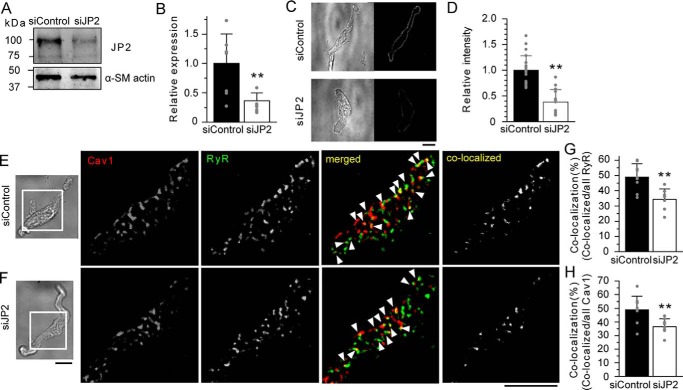
**JP2 mediates the molecular coupling of RyR and Cav1 in MASMCs.** *A,* Western blotting analyses were performed using the protein lysate (30 μg) from mouse mesenteric artery tissues treated with siRNA for 4 days. The band density of JP2 was normalized to that of α-SM actin (JP2/α-SM actin). The ratio of siJP2 was normalized to that of siControl. *B,* summarized effects of siControl and siJP2 on JP2 protein expression in the mesenteric artery. *C,* representative transmitted light and immunofluorescent images of mMASMCs treated with siControl or siJP2. Myocytes were labeled with an anti-JP2 antibody and observed using a confocal microscope. *D,* summarized effects of siControl (*n* = 23) and siJP2 (*n* = 14) on JP2 expression estimated by fluorescent intensity. *E* and *F,* RyR and Cav1 in mMASMCs treated with siControl (*E*) or siJP2 (*F*) were labeled with the specific antibody and observed under a TIRF microscope. Fluorescent signals corresponding to Cav1, RyR, and their co-localization are colored in *red*, *green*, and *yellow* (denoted by *arrowheads* in *merged*), respectively. The *yellow* puncta in *co-localized* indicate only the overlapping signals of Cav1 and RyR. *G* and *H,* the co-localization ratio of RyR (*G*) or Cav1 (*H*) particles in myocytes treated with siControl (*n* = 9) or siJP2 (*n* = 8). *, *p* < 0.05; **, *p* < 0.01; the Student's *t* test. *Scale bars* indicate 10 μm (*C*, *E*, and *F*).

In the next series of experiments, the knockdown effects of JP2 by siRNA were examined to assess the significance of JP2 in molecular coupling between Cav1 and RyR. The mesenteric arterial tissues of mice were organ-cultured for 4 days and concomitantly treated with control siRNA (siControl) or siRNA specific for JP2 (siJP2) using reversible permeabilization procedures (30). Western blotting and immunostaining analyses revealed that siJP2 significantly decreased JP2 protein expression in mesenteric arterial tissues and mMASMCs (Fig. 6, *A–D*).

In corresponding TIRF images of single myocytes isolated after the knockdown, fluorescent particles of RyR often co-localized with those of Cav1 in myocytes incorporated with siControl or siJP2 (Fig. 6, *E* and *F*), in a similar manner to that shown in Fig. 3. However, the ratio of co-localization was significantly lower in siJP2-treated myocytes than in siControl (for RyR, siControl, 48.9 ± 8.8%, *n* = 9; siJP2, 34.4 ± 6.9%, *n* = 8, *p* < 0.01; for Cav1, siControl, 48.9 ± 9.9%; siJP2, 36.6 ± 5.7%; *p* < 0.01, Fig. 6, *G* and *H*), although the number of fluorescent particles of Cav1 and JP2 was not altered (Table S1). These results provide additional evidence for JP2 connecting Cav1 and RyR and, thus, linking caveolae to the SR element.

### JP2 facilitates the efficiency of Ca^2+^ sparks forming STOCs.

**Figure 7.**
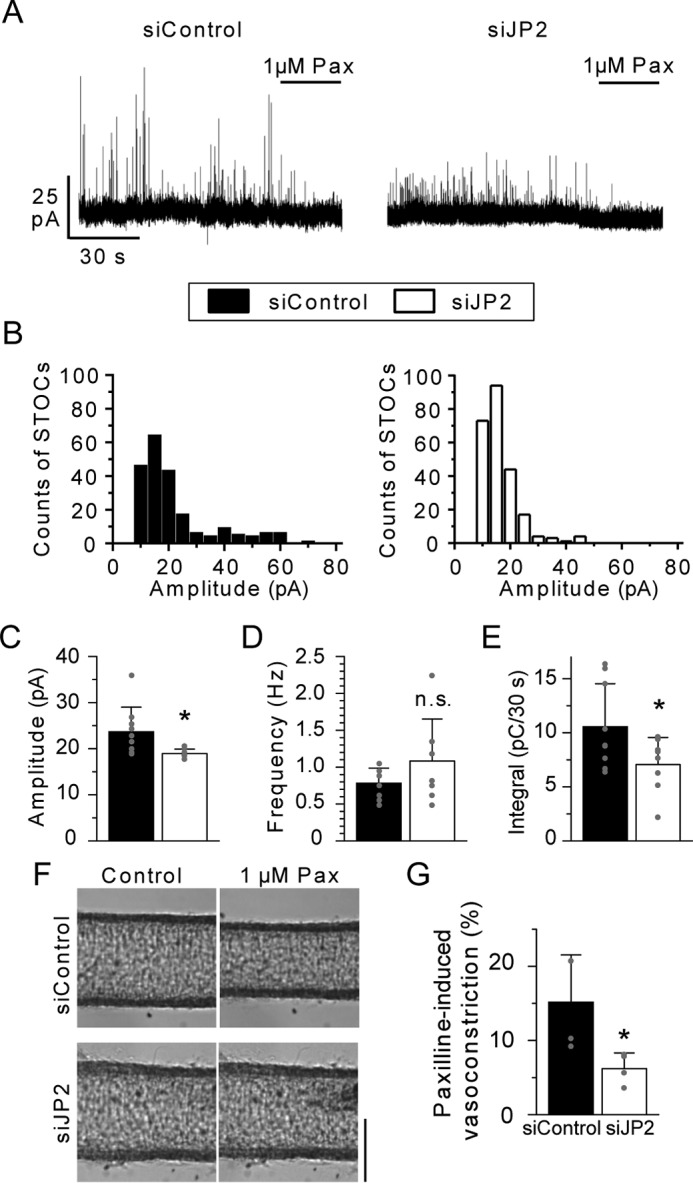
**JP2 facilitates the efficiency of STOCs in mMASMCs.** *A,* STOCs were recorded in siControl and siJP2-treated myocytes at a holding potential of −20 mV. STOCs were completely blocked by 1 μm paxilline (*Pax*). *B,* distribution histogram of STOC events *versus* their peak amplitude. *C–E,* summary of the mean STOC amplitude, frequency and integral for 30 s in myocytes treated with siControl (9 cells from 6 mice) or siJP2 (8 cells from 7 mice). *F,* images of vasocontractions caused by 1 μm Pax. The averaged diameter (μm) for: siControl, 125.7 ± 9.2 (Control) and 116.8 ± 10.0 (1 μm Pax); siJP2, 133.5 ± 14.6 (Control) and 130.5 ± 13.1 (1 μm Pax). *G,* summarized data of 1 μm Pax-induced decreases in the diameter of mesenteric arteries treated with siControl (*n* = 4 from 3 mice) and siJP2 (*n* = 4 from 3 mice). *, *p* < 0.05; **, *p* < 0.01; the Student's *t* test. The *scale bar* indicates 100 μm in *F. n.s*., not significant.

The proposed molecular complex may constitute a Ca^2+^ signaling pathway functionally distinct from those established in striated muscles. Thus, STOCs were measured as the results of functional coupling between Ca^2+^ sparks and BK_Ca_ channel activity at a holding potential of −20 mV in mMASMCs under the whole cell voltage-clamp mode (Fig. 7*A*). Single mMASMCs were isolated from tissues pre-treated with siControl or siJP2 for 4 days by reversible permeabilization. The principal results obtained were summarized as amplitude histograms of STOCs in Fig. 7*B*. The averaged STOC amplitude was significantly smaller in siJP2-treated mMASMCs than in siControl-treated myocytes (siControl, 23.7 ± 5.3 pA, *n* = 9; siJP2, 18.9 ± 1.0 pA, *n* = 8; *p* < 0.05, Fig. 7*C*). The averaged frequency was not significantly different between siControl- and siJP2-treated myocytes (siControl, 0.78 ± 0.20 Hz, *n* = 9; siJP2, 1.08 ± 0.57 Hz, *n* = 8; *p* > 0.05, Fig. 7*D*). The average value of STOCs integrated for 30 s was significantly smaller in siJP2-treated mMASMCs than in siControl-treated myocytes (siControl, 10.6 ± 4.0 pC, *n* = 9; siJP2, 7.1 ± 2.5 pC, *n* = 8; *p* < 0.05, Fig. 7*E*). Although the averaged STOC amplitude and the integrated STOCs were reduced by the treatment with siJP2, the whole-cell BK_Ca_ channel current density (Fig. S4) and amplitude of Ca^2+^ sparks were not affected (Fig. S5). In contrast, the frequency and area at which Ca^2+^ sparks spread (FWHM) were significantly increased, and the decay (*t*_½_ and FDHM) was prolonged by siJP2 (Fig. S5*D*). These results are consistent with previous findings that demonstrated enhanced RyR activity and reduced Na^+^/Ca^2+^-exchanger activity in JP2 knockdown cardiac myocytes (31–33). These results suggest that the knockdown of JP2 attenuates proximal coupling between BK_Ca_ channels and RyR, thereby decreasing the efficiency with which localized Ca^2+^ spark signals are converted to cellular STOC signals, *i.e.* Ca^2+^ derived from Ca^2+^ sparks did not sufficiently reach nearby BK_Ca_ channels.

To examine the contribution of JP2 to muscle contraction, the pressure myography measurements were performed in tissue preparations. The diameter of mouse mesenteric arteries pretreated with siControl or siJP2 was measured before and after the BK_Ca_ channel block (Fig. 7*F*). Contractions elicited by 1 μm paxilline were significantly weaker in siJP2-treated arteries than in those treated with siControl (siControl, 15.2 ± 6.4%; siJP2, 6.2 ± 2.1%, *n* = 4, *p* < 0.05, Fig. 7*G*). In contrast, there was no effect of siJP2 on 40 mm KCl-induced constrictions (Fig. S6*A*). These experiments were performed using artery segments, which had a similar maximal passive diameter under fully relaxed conditions (Fig. S6*B*). Taken together, it can be suggested that the knockdown of JP2 reduced the contribution of STOCs to the regulation of muscle tone and, thus, the susceptibility of resting tone to paxilline.

## Discussion

In skeletal and cardiac myocytes, JP1 and JP2 localize on the T-tubular membrane to organize JMC by linking various molecules such as ion channels (VDCC (34, 35), TRPC3 (36), and RyR2 (32)) and scaffolding proteins (Cav3 (37) and BIN-1 (bridging integrator-1) (23)). On the other hand, SMCs lack the T-tubular system. The functions of caveolae as a platform for E-C coupling (11) and STOC generation have instead been suggested in SMCs (12, 18). In the present study, we found that a key tethering molecule, JP2, bound with Cav1 (Figs. 1 and 2) and BK_Ca_ channels (Fig. 5 and Fig. S2). These molecular interactions, as well as that between JP2 and RyR (38), may play a critical role in the formation of the Ca^2+^ signaling pathway, which functionally connect BK_Ca_ channels in caveolae to RyR in junctional SR in VSMCs.

Co-IP and single molecular imaging analyses (Fig. 1) revealed the interaction between JP2 and Cav1 in mMASMCs. Moreover, BiFC assays based on a series of deletion/truncated mutants provided evidence to show that the 271–290 residues, particularly ^286^TTTET^290^, in the joining region of JP2 were important for the interaction with Cav1. Because this region contains many hydrophilic amino acids, JP2 and Cav1 may bind together through their regions facing the cytosol. We also demonstrated the molecular coupling of JP2 with BK_Ca_ channels, which preferentially localize in caveolae (12, 28), using co-IP and TIRF imaging (Fig. 5, Fig. S2). BiFC assays revealed that the 271–290 residues in the JP2 joining region were also an essential site for binding with BK_Ca_ channels as well as Cav1 (Fig. S2). The joining region also contains some interaction domains for other ion channels, such as VDCC (Cav1.1 and Cav1.2) (34, 35), TRPC3 (36), and RyR2 (32). In smooth muscle tissues, all three RyR isoforms are expressed, and the relative proportions of this expression are tissue- and species-dependent (39). We think that JP2 mainly interact with RyR2 in mesenteric artery smooth muscle cells because (i) JP2 does not bind to RyR1 (40), (ii) RyR2 binds to JP2 (23, 32), and (iii) Ca^2+^ spark-STOCs activity is rather high in mesenteric artery smooth muscle cells in the RyR3^−/−^ mouse (41). Therefore, JP2 appears to be the basis for a number of macromolecular complexes by accumulating Ca^2+^-related molecules in SMCs.

In the present study, the disruption of caveolae by MβCD resulted in: 1) a decrease in Cav1-JP2 co-localization (Fig. 1), but 2) no change in JP2-RyR co-localization (Fig. 3), and 3) a decrease in Cav1-RyR co-localization (Fig. 3). These results suggest that the caveolar structure mainly facilitates the interaction with Cav1-JP2. Moreover, caveolar disruption/abolishment by MβCD or the Cav1 gene deficiency, respectively, also significantly reduced JP2-BK_Ca_ channel coupling (Fig. 5). Cav1 facilitates this coupling by accumulating JP2 and BK_Ca_ channels (12, 28) within the caveolar structure. The present results are consistent with our previous findings showing that the STOC frequency was significantly lower in MASMCs from Cav1^−/−^ mice than in those from WT and that the occurrence of Ca^2+^ sparks was not altered (12). Thus, the significance of the caveolar structure in formation of the Ca^2+^-signaling pathway in SMCs may be explained by two factors. The invagination of caveolae into the cytosol shortens the distance between PM and SR and allows JP2 molecules to more easily facilitate functional coupling between RyR2 in SR and BK_Ca_ channels in PM. The interaction between JP2 and RyR2 and its functional importance have been shown in cardiac myocytes (23, 32). Our imaging analyses revealed the co-localization of Cav1-RyR in mMASMCs. The results from experiments using siJP2 (Fig. 6) clearly showed that JP2 was responsible for connecting RyR and Cav1 in the Ca^2+^-signaling pathway. In addition, related signal molecules in PM, such as BK_Ca_ channels and VDCC, accumulated in the caveolar structure and were primed to interact with one another and also with RyR for functional coupling in Ca^2+^ microdomains.

The spatio-positional relationships between RyR and BK_Ca_ channels (42) or scaffold proteins (43, 44) have only been reported using immunochemical staining in formalin-fixed myocytes, regardless of muscle types. In contrast, our molecular imaging of primary cultured mMASMCs, in which JP2 or Cav1 labeled with mCherry was expressed, enabled us to map the positional relationships between Ca^2+^ spark-generating sites and JP2 or Cav1 molecules (Fig. 4). It is especially notable that these measurements of the distance between spark sites and JP2 or Cav1 is performed in live imaging. Our results revealed that almost 50% of Ca^2+^ spark-generating sites localized within mCherry-JP2 particles (58.3%) or mCherry-Cav1 particles (39.7%). By taking the expression efficiency of mCherry-labeled JP2 or Cav1 in mMASMCs into account, Ca^2+^ spark-generating sites distant from mCherry particles may be located close to non-mCherry–tagged, native JP2 or Cav1 (12, 25). As a consequence, we may concretely conclude that the majority of Ca^2+^ sparks occur in JMCs bridged by JP2.

Based on analyses performed with an electron microscope or super-resolution microscope, Cav1 (45), JP2 (44), and RyR (46) form clusters as large as 200 nm or more in diameter (47). The mean radius values for adjacent mCherry-JP2 and mCherry-Cav1 complexes were 455 and 368 nm, respectively (Fig. 4). These mCherry particles may reveal functional molecular clusters. Previous studies reported that a Ca^2+^ spark spreads over an area of ∼500 nm in radius and increases [Ca^2+^]*_i_* up to 3.5 μm or higher in some SMCs (42, 48, 49). JP2, Cav1, BK_Ca_ channels, and other molecules were co-localized within ∼400 nm of Ca^2+^-spark sites (Fig. 4). Thus, these molecules will be exposed to high [Ca^2+^]*_i_* provided by Ca^2+^-spark events. A previous study reported that BK_Ca_ channels, which localize within 150∼300 nm of a Ca^2+^-spark site, may be strongly activated in airway SMCs (49). Our results provide evidence to suggest that a distinct molecular complex composed of Cav1, JP2, and junctional SR significantly facilitate the efficiency of functional coupling between RyR and BK_Ca_ channels in the structure.

The present results also revealed new and functionally important roles for JP2 in STOCs in siJP2-treated mMASMCs. siJP2 had no effect on functional BK_Ca_ channel expression or the amplitude of Ca^2+^ sparks, but significantly increased the frequency of Ca^2+^ sparks (Figs. S4 and S5). Because JP2 stabilizes RyR and reduces Ca^2+^ leakage in cardiac myocytes (31, 32), decreases in JP2 levels may have increased the spontaneous opening of RyR in mMASMCs. Despite this increase in Ca^2+^ spark frequency, JP2 knockdown did not significantly change STOC frequency, but, importantly, decreased the averaged STOC amplitude and integrated STOCs (Fig. 7). Small STOCs may be generated by Ca^2+^ sparks that occur away from PM (>1 μm) (50). Therefore, BK_Ca_ channels may be exposed to a larger number of Ca^2+^ sparks, which occur not close to BK_Ca_ channels in JP2-knocked down myocytes. This may be the reason why frequency did not decrease in siJP2-treated myocytes, although BK_Ca_-RyR coupling was weakened by the JP2 knockdown. In this case, the amount of Ca^2+^ reached from Ca^2+^ sparks to BK_Ca_ channels may have been smaller, leading to the decreased amplitude of STOCs in Fig. 7. Thus, JP2 knockdown reduced coupling efficiency between BK_Ca_ channels and RyR. A recent study showed that microtubule structures support peripheral coupling between BK_Ca_ channels and RyR in SMCs (51). Further studies are needed to elucidate the relationship between microtubules and the Cav1-JP2 interaction.

Although Cav3 is the muscle isoform among the three Cav isoforms, the predominant Cav isoform expressed in SMCs, including mMASMCs, has been reported as Cav1 (52) (Fig. S1). The physiological impact of the Cav1-JP2 interaction in the caveolae of MASMCs may be more significant with respect to Ca^2+^ handing than those of Cav3-JP1 (34) and Cav3-JP2 (37) in skeletal or cardiac muscles. Cav3 in skeletal and cardiac myocytes mainly forms caveolae in the extra T-tubular PM (53), whereas a molecular interaction between JP2 and Cav3 in T-tubules has also been suggested (38). The Cav1-BK_Ca_ channel interaction significantly facilitates the accumulation of BK_Ca_ channels in caveolae, in which BK_Ca_ channels form molecular complexes with VDCC (12) (see Fig. S7). Thus, the Cav1-JP2 interaction in SMCs provides a distinct structural basis essential for the formation of the Ca^2+^-signaling pathway in JMC and for most efficient signal coupling between Ca^2+^ sparks and STOCs to regulate membrane potential and, consequently, muscle tone. In addition, the novel Cav1-JP2 interaction and related molecular complex formation are presumably responsible for the Ca^2+^-induced Ca^2+^-release mechanism triggered by action potentials in mMASMCs (11, 12). The physiological impact of the interaction between the Cav1 and JP family (JP3) also needs to be considered in nonmuscle tissues because the co-expression of this pair of molecules has been reported in pancreatic β-cells (54, 55); however, the functional significance of this interaction remains unclear.

In summary, JP2 binds to three key molecules: Cav1, BK_Ca_ channel, and RyR, and connects them to functionalize a molecular complex in the site of Ca^2+^ microdomains from caveolae and SR in JMC (Fig. S7). The major physiological function of the Cav1-JP2 interaction in SMCs appears to differ from that of Cav3-JP1/JP2 in striated muscles. This distinct molecular complex in the JMCs of SMCs enable the effective conversion of Ca^2+^ sparks to cellular electrical signals, *i.e.* STOCs and membrane hyperpolarization. More generally, due to these structural and functional mechanisms essentially provided by the Cav1-JP2 interaction, Ca^2+^-sensitive signal molecules assembled in caveolae and in the site of Ca^2+^ microdomains can obtain sufficient Ca^2+^ without affecting other signaling pathways (56). This highly localized compartmentalization of Ca^2+^ signaling based on Cav1-JP2 interactions is critical for well-regulated SM tissue functions, including the regulation of vascular tone and blood pressure. Furthermore, the Cav-JP interaction may provide a novel structural/functional basis for reciprocal cross-talk between molecules that accumulate in the caveolae of PM and those in the ER, even in nonmuscular cells.

## Experimental procedures

### Animals

Mesenteric arteries were dissected from male mice or rats (C57BL/6, 8–12 weeks and Wistar/ST, 8–12 weeks, respectively, SLC, Hamamatsu, Japan) (12). Animals were anesthetized by the inhalation of isoflurane (Baxter, Deerfield, IL) and killed by cervical dislocation. To isolate mMASMCs, tissues were incubated in Ca^2+^/Mg^2+^-free Hanks' solution: (mm) 137 NaCl, 5.4 KCl, 0.17 Na_2_HPO_4_, 0.44 KH_2_PO_4_, 4.2 NaHCO_3_, and 5.6 glucose, containing 0.4% collagenase (Wako, Osaka, Japan) and 0.1% papain (Sigma) at 37 °C for 40 min. After the incubation, tissues were washed in Ca^2+^/Mg^2+^-free Hanks' solution and treated by gentle agitation with a glass pipette. Single mMASMCs were suspended in M-199 (Sigma) supplemented with 2% heat-inactivated FBS (Sigma), 20 units/ml of penicillin (Wako), and 20 μg/ml of streptomycin (Wako), and then cultured on glass-bottomed dishes for 24–48 h. All experiments were approved by the Ethics Committee of Nagoya City University and conducted in accordance with the Guide for the Care and Use of Laboratory Animals of the Japanese Pharmacological Society.

### Plasmid constructs and transfection

The full-length cDNAs encoding JP2 (NM_020433.4) and Cav1 (NM_001753) were labeled with fluorescent proteins (using pEYFP-C1, pAcGFP1-C1, or pmCherry-C1 vectors from Clontech Laboratories, Mountain View, CA) at the N terminus. BKα (NM_002247) was labeled with fluorescent proteins (using the pECFP-N1 vector from Clontech Laboratories) at the C terminus. In BiFC analyses, constructs were labeled by fragments of the N (1–173: VN173) or C (155–238: VC155) termini of Venus (22). pBiFC-VN173 and pBiFC-VC155 were gifts from Chang-Deng Hu (Addgene plasmid numbers 22010 and 22011, respectively). All constructs were confirmed by DNA sequencing. Primary-cultured myocytes isolated from mouse mesenteric arteries were transiently transfected with mCherry-labeled cDNA using Lipofectamine 2000 (Invitrogen). Experiments were performed 24–48 h after transfection.

### Reversible permeabilization

siRNA was introduced into mesenteric arteries using a reversible permeabilization procedure (30). In brief, mesenteric arteries were incubated at 4 °C for 30 min in the following solution (mm): 120 KCl; 2 MgCl_2_, 10 EGTA, 5 Na_2_ATP, and 20 TES, pH 6.8. Thereafter, tissues were placed in a solution containing 10 μm siRNA (pre-designed Stealth RNAi^TM^ for the knockdown of mouse JP2: Jph2MSS226908, 5′-AGU ACC GCC ACA AUG UGC UGG UCA A-3′ or Medium GC Duplex number 3 for siControl, Invitrogen) at 4 °C for 90 min and then in 10 μm siRNA-containing solution with 10 mm MgCl_2_ at 22 °C for 30 min. Permeabilization was reversed by placing the tissue at 22 °C for 30 min in MOPS-buffered physiological solution containing (mm): 140 NaCl, 5 KCl, 10 MgCl_2,_ 5 glucose, and 2 MOPS, pH 7.1. Ca^2+^ was gradually increased over a 45-min period from nominally Ca^2+^ free to 0.01, 0.1, and 1.8 mm. Following the reversible permeabilization procedures, the mesenteric artery was organ-cultured for 4–5 days in M-199 with 2% FBS. After 2 days, this culture medium was refreshed.

### TIRF imaging

Two-dimensional Ca^2+^ imaging and single-molecular imaging were obtained using a TIRF imaging system (Nikon, Tokyo, Japan), which consists of a fluorescent microscope (ECLIPSE TE2000-U; Nikon), objective lens (CFI Plan Apo TIRF ×60/1.45, oil immersion; Nikon), EM-CCD camera (C9100–12; Hamamatsu Photonics, Hamamatsu, Japan), and AQUACOSMOS software (version 2.6; Hamamatsu Photonics) (12, 25). Regarding Ca^2+^-spark measurements, myocytes were loaded with 10 μm fluo-4 acetoxymethyl ester (fluo-4/AM; Invitrogen-Molecular Probes). Fluo-4/AM was excited with a 488-nm argon laser (Coherent, Santa Clara, CA), and emissions were collected using a dual color filter cube (DM 480–495/538–556 nm, BA 505–530/570–660 nm, Omega Optical, USA). To observe Ca^2+^ sparks in a myocyte, 40 mm KCl HEPES-buffered solution (40 KCl), which contained (mm) 102.9 NaCl, 40 KCl, 2.2 CaCl_2_, 1.2 MgCl_2_, 14 glucose, and 10 HEPES (pH 7.4 with NaOH), was applied. Fluorescent signals are described as *F*/*F*_0_, where *F* is the averaged fluorescence intensity in the TIRF area during measurements, and *F*_0_ is the baseline *F* value obtained as the average intensity of the regions of interest (2 μm in diameter). Ca^2+^ images were collected at 27.3-ms exposure intervals. After Ca^2+^ spark measurements, mCherry-labeled proteins were excited with a 543-nm He/Ne laser (Coherent). When Ca^2+^ sparks occurred within 454.9 nm (mean values of the radius of mCherry-JP2) or 368 nm (distance of the peak points of the nearest mCherry spots) (see Fig. 4), these two fluorescence signals were considered to co-localize. These fluorescent particles occupied only 0.05% of the surface area attached to the bottom of the culture dishes. This weak expression of mCherry indicated that our experimental maneuver did not cause the overexpression of transfected genes, and may be used to detect single fluorescent molecules in a high signal to noise ratio (25). Immunolabeling images were collected by exposure for 465 ms. The resolution of images was 178 nm per pixel (*x-y*) and less than 200 nm (z). All experiments were performed at room temperature (25 °C).

### FRET analysis

E_FRET_ was evaluated based on the acceptor photobleaching method, in which the emission of the donor fluorophore was compared before and after the photobleaching of the acceptor (12). The fluorescence of YFP was photobleached using a mercury lamp (100 W, C-SHG1; Nikon) and G-2A filter cube (Ex 510–560/DM575/BA590; Nikon) for 2.5 min. After CFP and YFP fluorescence was detected, a 405-nm blue diode laser (Coherent) or 488-nm laser was used for excitation. CFP-HQ (DM450/BA460–510; Nikon) and YFP-HQ (DM510/BA520–560; Nikon) filter cubes were used for the collection of emission signals. E_FRET_ was calculated as the percentage increase in CFP emission after YFP photobleaching, as described previously (12).

### Confocal imaging

Confocal images were obtained using a laser scanning confocal fluorescent microscope (A1R, Nikon) equipped with a fluorescent unit (ECLIPSE Ti), objective lens (Plan Apo ×60 1.40 NA, oil immersion), and NIS Elements software (version 3.10; Nikon). In BiFC assay (Fig. 2*D*), images were acquired using ×20 objective lens (2048 × 2048 pixels, 0.31 μm/pixel). The averaged fluorescence intensity of complimented Venus in the images was divided by that of the Hoechst signals. The Venus/Hoechst ratio of the mutants ([*4-ii*], [*4-iii*], and [*5*]) was normalized to that of [*4-i*].

### Immunocytochemistry

Single MASMCs were fixed with 4% paraformaldehyde in phosphate-buffered saline (PBS). These cells were treated with 0.2% Triton X-100, 2% bovine serum albumin (BSA, Sigma), and a goat polyclonal anti-JP2 antibody (1:50 dilution; γ-15, Santa Cruz Biotechnology, Dallas, TX), rabbit polyclonal anti-Cav1 antibody (1:1000 dilution; Sigma), rabbit polyclonal anti-BKα antibody (APC-107, Alomone Laboratories, Jerusalem, Israel), or mouse monoclonal anti-RyR antibody (clone 34C; Sigma, 1:1000) at 4 °C for 12 h. After washing repeatedly, the preparations were treated with Alexa 488- or Alexa 405-conjugated secondary antibodies (1:1000, Molecular Probes) at room temperature for 1 h. Fluorescently labeled cells were observed using a confocal imaging system or the TIRF imaging system described above. Alexa 405 and Alexa 488 were excited with the blue diode and argon laser, respectively. The emissions of Alexa 405 and Alexa 488 were collected using CFP-HQ and YFP-HQ filter cubes, respectively.

The specificity of the anti-JP2 antibody (γ-15, Santa Cruz) was proved by the following data: (i) the antibody detected a single band that corresponded to the predicted molecular mass of JP2 (∼100 kDa) in a Western blot analysis (Fig. 1*A*), (ii) the antibody detected JP2 expression along with PM in immunostaining experiments (Fig. 1*B*), and (iii) siJP2 clearly decreased the band density (Fig. 6, *A* and *B*) as well as fluorescent signal (Fig. 6, *C* and *D*). This antibody has been used in many other studies (57–59). The anti-BKα antibody and anti-RyR antibody have also been used in other studies (51).

The specificity of the anti-Cav1 antibody was validated in experiments using mesenteric artery SM cells from Cav1^−/−^ mice. The antibody clearly detected Cav1 on PM in WT mouse, whereas no signal was noted in myocytes from Cav1^−/−^ mouse (data not shown).

### In situ PLAs

To clarify whether JP2 and Cav1 co-localize within 40 nm of mMASMCs, PLAs were performed using a PLA kit (Duolink, Sigma) (60). Single mMASMCs were fixed with 4% paraformaldehyde in PBS and treated with 0.2% Triton X-100. Cells were then labeled with anti-JP2 and anti-Cav1 antibodies in PBS containing 2% BSA at 4 °C for 12 h. After washing repeatedly, cells were incubated in a humidified chamber at 37 °C for 1 h with secondary anti-goat PLUS and anti-rabbit MINUS PLA probes and then washed in Duolink Wash Buffer A. The preparations were incubated in ligation-ligase solution at 37 °C for 30 min in a humidifier chamber and then washed repeatedly in Wash Buffer A. Samples were incubated in Amplification Polymerase solution at 37 °C for 100 min in a humidifier chamber and then washed repeatedly in Duolink Wash Buffer B. Fluorescence images were observed using a confocal imaging system. When two PLAs probes were within 40 nm, positive signals (green puncta) were generated. The excitation of fluorescent puncta was illuminated at 488 nm. Negative control experiments were performed using mMASMCs from Cav1^−/−^ mice.

### Co-IP and Western blotting

Co-IP was performed using the Pierce Co-Immunoprecipitation Kit (number 26149) according to the experimental manual supplied by Thermo Scientific (Waltham, MA) (22). The rat mesenteric artery was lysed in IP lysis/wash buffer with protease inhibitor mixture (Sigma). Precleared whole lysates were incubated with AminoLinkPlus Coupling Resin or Control Agarose Resin slurry with which 10 μg of anti-Cav1 was immobilized, washed with IP lysis/wash buffer, eluted with elution buffer, and subjected to SDS-PAGE (10%). In Western blot analysis, 30 μg of total protein was applied to the gels. The blots were incubated with anti-JP2 antibodies and then incubated with anti-goat horseradish peroxidase-conjugated IgG (Chemicon International, Temecula, CA). Images were obtained using an enhanced chemiluminescence detection system (Amersham Biosciences, Piscataway, NJ) and analyzed by a LAS-3000 device (Fujifilm, Tokyo, Japan). Full western blot images are presented in Fig. S8.

### Electrophysiological recording

Electrophysiological studies were performed using a whole-cell voltage-clamp technique with a CEZ-2400 amplifier (Nihon Kohden, Tokyo, Japan), analog-digital converter (DIGIDATA 1440A; Axon Instruments, Foster City, CA), and pCLAMP software (version 10.3; Axon Instruments) (12). In recordings of STOCs, the pipette solution contained (mm): 140 KCl, 4 MgCl_2_, 10 HEPES, 0.05 EGTA, and 2 Na_2_ATP (pH 7.2 with KOH). In BK_Ca_ channel current measurements, the pipette solution contained (mm): 140 KCl, 2.8 MgCl_2_, 10 HEPES, 2 Na_2_ATP, 5 EGTA, and 3.19 CaCl_2_ (*p*Ca 6.5). pH was adjusted to 7.2 with KOH. The extracellular solution (normal HEPES-buffered solution) had an ionic composition of (mm): 137 NaCl, 5.9 KCl, 2.2 CaCl_2_, 1.2 MgCl_2_, 14 glucose, and 10 HEPES. pH was adjusted to 7.4 with NaOH.

### Pressure myography

Endothelium-denuded mouse mesenteric arteries (4th branch) were cannulated at each end in a perfusion chamber and loaded with 60 mm Hg. They were placed in standard Krebs solution containing (in mm): 112 NaCl, 4.7 KCl, 2.2 CaCl_2_, 1.2 MgCl_2_, 25 NaHCO_3_, 1.2 KH_2_PO_4_, and 14 glucose, aerated with 95% O_2_-5% CO_2_ to obtain pH 7.4. The arterial diameter (*D*) was measured using a CCD camera. Vasoconstriction was calculated as follows: (1 − *D*/*D*_max_), where *D*_max_ is the diameter measured in the presence of Ca^2+^- and Mg^2+^-free Krebs solution containing (mm): 112 NaCl, 4.7 KCl, 25 NaHCO_3_, 1.2 KH_2_PO_4_, 14 glucose, and 1.1 EGTA, aerated with 95% O_2_, 5% CO_2_ to obtain pH 7.4.

### Data notation and statistical analysis

Pooled data are shown as the mean ± S.D. with the numbers of samples or cells. The significance of differences between two groups was evaluated using the Student's *t* test after the application of the *F*-test. Data from more than two groups were compared using a one-way analysis of variance (ANOVA), followed by Tukey's test. Two-way ANOVA was applied for the analysis of FRET data in WT and Cav1^−/−^ (Fig. 5*J*). In all cases, *p* values <5% (*p* < 0.05) were considered to be significant. The mean radius of mCherry particles was calculated by fitting histograms with the Lorenz curve.

### Drugs

Reagents were obtained from Wako Ltd., except for MβCD (Sigma), EGTA, HEPES (Dojin, Kumamoto, Japan), and paxilline (Alomone). All hydrophobic compounds were dissolved in dimethyl sulfoxide (DMSO) at a concentration of 1–10 mm as a stock solution. We confirmed that up to 0.1% of DMSO did not affect any of the response patterns.

## Author contributions

T. S., Y. S., H. Y., H. T., and Y. I. conceptualization; T. S., Y. S., H. Y., and Y. I. resources; T. S. data curation; T. S., Y. S., and Y. I. formal analysis; T. S., Y. S., H. Y., and Y. I. funding acquisition; T. S., Y. S., H. Y., H. T., and Y. I. validation; T. S. and Y. S. investigation; T. S., Y. S., H. Y., and Y. I. visualization; T. S., Y. S., H. Y., H. T., and Y. I. methodology; T. S., Y. S., and Y. I. writing-original draft; Y. S., H. Y., and Y. I. supervision; Y. S., H. Y., H. T., and Y. I. writing-review and editing; H. Y. and Y. I. project administration.

## Supplementary Material

Supporting Information

## Footnotes

2 The abbreviations used are:

JP: junctophilin
BK_Ca_: large-conductance Ca^2+^-activated K^+^
PM: plasma membrane
RyR: ryanodine receptor
ER/SR: endo/sarcoplasmic reticulum
SMC: smooth muscle cell
Cav1: caveolin-1
JP2: junctophilin-2
JMC: junctional membrane complex
VDCC: voltage-gated Ca^2+^ channel
VSMC: vascular smooth muscle cell
STOC: spontaneous transient outward current
TIRF: total internal reflection fluorescence
PLA: proximity ligation assay
MβCD: methyl-β-cyclodextrin
BiFC: bimolecular fluorescent complementation
FWHM: full width at half-maximum
Cav1^−/−^: Cav1 knockout mice
E_FRET_: FRET efficiency
Pax: paxilline
mMASMC: mouse mesenteric artery SMC
co-IP: co-immunoprecipitation
TES: 2-{[2-hydroxy-1,1-bis(hydroxymethyl)ethyl]amino}ethanesulfonic acid
ANOVA: analysis of variance
CFP: cyan fluorescent protein
YFP: yellow fluorescent protein.

## References

[1] Berridge M. J. (2006) Calcium microdomains: organization and function. Cell Calcium 40, 405–412 10.1016/j.ceca.2006.09.002 17030366

[2] Takeshima H., Komazaki S., Nishi M., Iino M., and Kangawa K. (2000) Junctophilins: a novel family of junctional membrane complex proteins. Mol. Cell 6, 11–22 10949023 10.1016/s1097-2765(00)00003-4

[3] Nishi M., Mizushima A., Nakagawara K., and Takeshima H. (2000) Characterization of human junctophilin subtype genes. Biochem. Biophys. Res. Commun. 273, 920–927 10.1006/bbrc.2000.3011 10891348

[4] Franzini-Armstrong C., and Protasi F. (1997) Ryanodine receptors of striated muscles: a complex channel capable of multiple interactions. Physiol. Rev. 77, 699–729 10.1152/physrev.1997.77.3.699 9234963

[5] Bers D. M. (2002) Cardiac excitation-contraction coupling. Nature 415, 198–205 10.1038/415198a 11805843

[6] Yamazawa T., Takeshima H., Sakurai T., Endo M., and Iino M. (1996) Subtype specificity of the ryanodine receptor for Ca^2+^ signal amplification in excitation-contraction coupling. EMBO J. 15, 6172–6177 10.1002/j.1460-2075.1996.tb01005.x 8947039 PMC452438

[7] Nishi M., Sakagami H., Komazaki S., Kondo H., and Takeshima H. (2003) Coexpression of junctophilin type 3 and type 4 in brain. Brain Res. Mol. Brain Res. 118, 102–110 10.1016/S0169-328X(03)00341-3 14559359

[8] Takeshima H., Hoshijima M., and Song L. S. (2015) Ca^2+^ microdomains organized by junctophilins. Cell Calcium 58, 349–356 10.1016/j.ceca.2015.01.007 25659516 PMC5159448

[9] Matsushita Y., Furukawa T., Kasanuki H., Nishibatake M., Kurihara Y., Ikeda A., Kamatani N., Takeshima H., and Matsuoka R. (2007) Mutation of junctophilin type 2 associated with hypertrophic cardiomyopathy. J. Hum. Genet. 52, 543–548 10.1007/s10038-007-0149-y 17476457

[10] Bennett H. J., Davenport J. B., Collins R. F., Trafford A. W., Pinali C., and Kitmitto A. (2013) Human junctophilin-2 undergoes a structural rearrangement upon binding PtdIns_(3,4,5)_P_3_ and the S101R mutation identified in hypertrophic cardiomyopathy obviates this response. Biochem. J. 456, 205–217 10.1042/BJ20130591 24001019 PMC3898329

[11] Hotta S., Yamamura H., Ohya S., and Imaizumi Y. (2007) Methyl-β-cyclodextrin prevents Ca^2+^-induced Ca^2+^ release in smooth muscle cells of mouse urinary bladder. J. Pharmacol. Sci. 103, 121–126 10.1254/jphs.SC0060213 17202744

[12] Suzuki Y., Yamamura H., Ohya S., and Imaizumi Y. (2013) Caveolin-1 facilitates the direct coupling between large conductance Ca^2+^-activated K^+^ (BK_Ca_) and Cav1.2 Ca^2+^ channels and their clustering to regulate membrane excitability in vascular myocytes. J. Biol. Chem. 288, 36750–36761 10.1074/jbc.M113.511485 24202214 PMC3868784

[13] Nelson M. T., Cheng H., Rubart M., Santana L. F., Bonev A. D., Knot H. J., and Lederer W. J. (1995) Relaxation of arterial smooth muscle by calcium sparks. Science 270, 633–637 10.1126/science.270.5236.633 7570021

[14] Yamazaki D., Tabara Y., Kita S., Hanada H., Komazaki S., Naitou D., Mishima A., Nishi M., Yamamura H., Yamamoto S., Kakizawa S., Miyachi H., Yamamoto S., Miyata T., Kawano Y., et al (2011) TRIC-A channels in vascular smooth muscle contribute to blood pressure maintenance. Cell Metab. 14, 231–241 10.1016/j.cmet.2011.05.011 21803293

[15] Bolton T. B., and Imaizumi Y. (1996) Spontaneous transient outward currents in smooth muscle cells. Cell Calcium 20, 141–152 10.1016/S0143-4160(96)90103-7 8889205

[16] Berkefeld H., Fakler B., and Schulte U. (2010) Ca^2+^-activated K^+^ channels: from protein complexes to function. Physiol. Rev. 90, 1437–1459 10.1152/physrev.00049.2009 20959620

[17] Cohen A. W., Hnasko R., Schubert W., and Lisanti M. P. (2004) Role of caveolae and caveolins in health and disease. Physiol. Rev. 84, 1341–1379 10.1152/physrev.00046.2003 15383654

[18] Cheng X., and Jaggar J. H. (2006) Genetic ablation of caveolin-1 modifies Ca^2+^ spark coupling in murine arterial smooth muscle cells. Am. J. Physiol. Heart Circ. Physiol. 290, H2309–2319 10.1152/ajpheart.01226.2005 16428350 PMC1698957

[19] Popescu L. M., Gherghiceanu M., Mandache E., and Cretoiu D. (2006) Caveolae in smooth muscles: nanocontacts. J. Cell Mol. Med. 10, 960–990 10.1111/j.1582-4934.2006.tb00539.x 17125599 PMC3933089

[20] Suzuki Y., Yamamura H., Ohya S., and Imaizumi Y. (2013) Direct molecular interaction of caveolin-3 with KCa1.1 channel in living HEK293 cell expression system. Biochem. Biophys. Res. Commun. 430, 1169–1174 10.1016/j.bbrc.2012.12.015 23237801

[21] Prakash Y. S., Thompson M. A., Vaa B., Matabdin I., Peterson T. E., He T., and Pabelick C. M. (2007) Caveolins and intracellular calcium regulation in human airway smooth muscle. Am. J. Physiol. Lung Cell. Mol. Physiol. 293, L1118–1126 10.1152/ajplung.00136.2007 17704188

[22] Suzuki Y., Ohya S., Yamamura H., Giles W. R., and Imaizumi Y. (2016) A new splice variant of large conductance Ca^2+^-activated K^+^ (BK) channel α subunit alters human chondrocyte function. J. Biol. Chem. 291, 24247–24260 10.1074/jbc.M116.743302 27758860 PMC5104946

[23] Jiang M., Zhang M., Howren M., Wang Y., Tan A., Balijepalli R. C., Huizar J. F., and Tseng G. N. (2016) JPH-2 interacts with Ca_i_-handling proteins and ion channels in dyads: contribution to premature ventricular contraction-induced cardiomyopathy. Heart Rhythm 13, 743–752 10.1016/j.hrthm.2015.10.037 26538326 PMC4762763

[24] Yamamura H., and Imaizumi Y. (2012) Total internal reflection fluorescence imaging of Ca^2+^-induced Ca^2+^ release in mouse urinary bladder smooth muscle cells. Biochem. Biophys. Res. Commun. 427, 54–59 10.1016/j.bbrc.2012.08.145 22975345

[25] Yamamura H., Ikeda C., Suzuki Y., Ohya S., and Imaizumi Y. (2012) Molecular assembly and dynamics of fluorescent protein-tagged single K_Ca_1.1 channel in expression system and vascular smooth muscle cells. Am. J. Physiol. Cell Physiol. 302, C1257–1268 10.1152/ajpcell.00191.2011 22301058

[26] Jaggar J. H., Porter V. A., Lederer W. J., and Nelson M. T. (2000) Calcium sparks in smooth muscle. Am. J. Physiol. Cell Physiol. 278, C235–256 10.1152/ajpcell.2000.278.2.C235 10666018

[27] Zhuge R., Fogarty K. E., Baker S. P., McCarron J. G., Tuft R. A., Lifshitz L. M., and Walsh J. V. Jr. (2004) Ca^2+^ spark sites in smooth muscle cells are numerous and differ in number of ryanodine receptors, large-conductance K^+^ channels, and coupling ratio between them. Am. J. Physiol. Cell Physiol. 287, C1577–1588 10.1152/ajpcell.00153.2004 15306542

[28] Alioua A., Lu R., Kumar Y., Eghbali M., Kundu P., Toro L., and Stefani E. (2008) Slo1 caveolin-binding motif, a mechanism of caveolin-1-Slo1 interaction regulating Slo1 surface expression. J. Biol. Chem. 283, 4808–4817 10.1074/jbc.M709802200 18079116

[29] Drab M., Verkade P., Elger M., Kasper M., Lohn M., Lauterbach B., Menne J., Lindschau C., Mende F., Luft F. C., Schedl A., Haller H., and Kurzchalia T. V. (2001) Loss of caveolae, vascular dysfunction, and pulmonary defects in caveolin-1 gene-disrupted mice. Science 293, 2449–2452 10.1126/science.1062688 11498544

[30] Muraki K., Iwata Y., Katanosaka Y., Ito T., Ohya S., Shigekawa M., and Imaizumi Y. (2003) TRPV2 is a component of osmotically sensitive cation channels in murine aortic myocytes. Circ. Res. 93, 829–838 10.1161/01.RES.0000097263.10220.0C 14512441

[31] van Oort R. J., Garbino A., Wang W., Dixit S. S., Landstrom A. P., Gaur N., De Almeida A. C., Skapura D. G., Rudy Y., Burns A. R., Ackerman M. J., and Wehrens X. H. (2011) Disrupted junctional membrane complexes and hyperactive ryanodine receptors after acute junctophilin knockdown in mice. Circulation 123, 979–988 10.1161/CIRCULATIONAHA.110.006437 21339484 PMC3056402

[32] Beavers D. L., Wang W., Ather S., Voigt N., Garbino A., Dixit S. S., Landstrom A. P., Li N., Wang Q., Olivotto I., Dobrev D., Ackerman M. J., and Wehrens X. H. (2013) Mutation E169K in junctophilin-2 causes atrial fibrillation due to impaired RyR2 stabilization. J Am. Coll. Cardiol. 62, 2010–2019 10.1016/j.jacc.2013.06.052 23973696 PMC3830688

[33] Wang W., Landstrom A. P., Wang Q., Munro M. L., Beavers D., Ackerman M. J., Soeller C., and Wehrens X. H. (2014) Reduced junctional Na^+^/Ca^2+^-exchanger activity contributes to sarcoplasmic reticulum Ca^2+^ leak in junctophilin-2-deficient mice. Am. J. Physiol. Heart Circ. Physiol. 307, H1317–1326 10.1152/ajpheart.00413.2014 25193470 PMC4217007

[34] Golini L., Chouabe C., Berthier C., Cusimano V., Fornaro M., Bonvallet R., Formoso L., Giacomello E., Jacquemond V., and Sorrentino V. (2011) Junctophilin 1 and 2 proteins interact with the l-type Ca^2+^ channel dihydropyridine receptors (DHPRs) in skeletal muscle. J. Biol. Chem. 286, 43717–43725 10.1074/jbc.M111.292755 22020936 PMC3243543

[35] Nakada T., Kashihara T., Komatsu M., Kojima K., Takeshita T., and Yamada M. (2018) Physical interaction of junctophilin and the CaV1.1 C terminus is crucial for skeletal muscle contraction. Proc. Natl. Acad. Sci. U.S.A. 115, 4507–4512 10.1073/pnas.1716649115 29632175 PMC5924888

[36] Woo J. S., Hwang J. H., Ko J. K., Kim D. H., Ma J., and Lee E. H. (2009) Glutamate at position 227 of junctophilin-2 is involved in binding to TRPC3. Mol. Cell. Biochem. 328, 25–32 10.1007/s11010-009-0070-0 19277847 PMC2990405

[37] Minamisawa S., Oshikawa J., Takeshima H., Hoshijima M., Wang Y., Chien K. R., Ishikawa Y., and Matsuoka R. (2004) Junctophilin type 2 is associated with caveolin-3 and is down-regulated in the hypertrophic and dilated cardiomyopathies. Biochem. Biophys. Res. Commun. 325, 852–856 10.1016/j.bbrc.2004.10.107 15541368

[38] Landstrom A. P., Beavers D. L., and Wehrens X. H. (2014) The junctophilin family of proteins: from bench to bedside. Trends Mol. Med. 20, 353–362 10.1016/j.molmed.2014.02.004 24636942 PMC4041816

[39] Wray S., and Burdyga T. (2010) Sarcoplasmic reticulum function in smooth muscle. Physiol. Rev. 90, 113–178 10.1152/physrev.00018.2008 20086075

[40] Phimister A. J., Lango J., Lee E. H., Ernst-Russell M. A., Takeshima H., Ma J., Allen P. D., and Pessah I. N. (2007) Conformation-dependent stability of junctophilin 1 (JP1) and ryanodine receptor type 1 (RyR1) channel complex is mediated by their hyper-reactive thiols. J. Biol. Chem. 282, 8667–8677 10.1074/jbc.M609936200 17237236

[41] Matsuki K., Kato D., Takemoto M., Suzuki Y., Yamamura H., Ohya S., Takeshima H., and Imaizumi Y. (2018) Negative regulation of cellular Ca^2+^ mobilization by ryanodine receptor type 3 in mouse mesenteric artery smooth muscle. Am. J. Physiol. Cell Physiol. 315, C1–C9 10.1152/ajpcell.00006.2018 29537866

[42] Lifshitz L. M., Carmichael J. D., Lai F. A., Sorrentino V., Bellvé K., Fogarty K. E., and ZhuGe R. (2011) Spatial organization of RYRs and BK channels underlying the activation of STOCs by Ca^2+^ sparks in airway myocytes. J. Gen. Physiol. 138, 195–209 10.1085/jgp.201110626 21746845 PMC3149436

[43] Wong J., Baddeley D., Bushong E. A., Yu Z., Ellisman M. H., Hoshijima M., and Soeller C. (2013) Nanoscale distribution of ryanodine receptors and caveolin-3 in mouse ventricular myocytes: dilation of t-tubules near junctions. Biophys. J. 104, L22–24 10.1016/j.bpj.2013.02.059 23746531 PMC3672889

[44] Jayasinghe I. D., Baddeley D., Kong C. H., Wehrens X. H., Cannell M. B., and Soeller C. (2012) Nanoscale organization of junctophilin-2 and ryanodine receptors within peripheral couplings of rat ventricular cardiomyocytes. Biophys. J. 102, L19–21 10.1016/j.bpj.2012.01.034 22404946 PMC3296050

[45] Fernandez I., Ying Y., Albanesi J., and Anderson R. G. (2002) Mechanism of caveolin filament assembly. Proc. Natl. Acad. Sci. U.S.A. 99, 11193–11198 10.1073/pnas.172196599 12167674 PMC123232

[46] Baddeley D., Jayasinghe I. D., Lam L., Rossberger S., Cannell M. B., and Soeller C. (2009) Optical single-channel resolution imaging of the ryanodine receptor distribution in rat cardiac myocytes. Proc. Natl. Acad. Sci. U.S.A. 106, 22275–22280 10.1073/pnas.0908971106 20018773 PMC2799702

[47] Wagner E., Lauterbach M. A., Kohl T., Westphal V., Williams G. S., Steinbrecher J. H., Streich J. H., Korff B., Tuan H. T., Hagen B., Luther S., Hasenfuss G., Parlitz U., Jafri M. S., Hell S. W., Lederer W. J., and Lehnart S. E. (2012) Stimulated emission depletion live-cell super-resolution imaging shows proliferative remodeling of T-tubule membrane structures after myocardial infarction. Circ. Res. 111, 402–414 10.1161/CIRCRESAHA.112.274530 22723297 PMC4219578

[48] Bao R., Lifshitz L. M., Tuft R. A., Bellvé K., Fogarty K. E., and ZhuGe R. (2008) A close association of RyRs with highly dense clusters of Ca^2+^-activated Cl^−^ channels underlies the activation of STICs by Ca^2+^ sparks in mouse airway smooth muscle. J. Gen. Physiol. 132, 145–160 10.1085/jgp.200709933 18591421 PMC2442178

[49] Zhuge R., Fogarty K. E., Tuft R. A., and Walsh J. V. Jr. (2002) Spontaneous transient outward currents arise from microdomains where BK channels are exposed to a mean Ca^2+^ concentration on the order of 10 μm during a Ca^2+^ spark. J. Gen. Physiol. 120, 15–27 10.1085/jgp.20028571 12084772 PMC2311396

[50] Ohi Y., Yamamura H., Nagano N., Ohya S., Muraki K., Watanabe M., and Imaizumi Y. (2001) Local Ca^2+^ transients and distribution of BK channels and ryanodine receptors in smooth muscle cells of guinea pig vas deferens and urinary bladder. J. Physiol. 534, 313–326 10.1111/j.1469-7793.2001.t01-3-00313.x 11454953 PMC2278703

[51] Pritchard H. A. T., Gonzales A. L., Pires P. W., Drumm B. T., Ko E. A., Sanders K. M., Hennig G. W., and Earley S. (2017) Microtubule structures underlying the sarcoplasmic reticulum support peripheral coupling sites to regulate smooth muscle contractility. Sci. Signal. 10, eaan2694 10.1126/scisignal.aan2694 28928237 PMC6328376

[52] Song K. S., Scherer P. E., Tang Z., Okamoto T., Li S., Chafel M., Chu C., Kohtz D. S., and Lisanti M. P. (1996) Expression of caveolin-3 in skeletal, cardiac, and smooth muscle cells: caveolin-3 is a component of the sarcolemma and co-fractionates with dystrophin and dystrophin-associated glycoproteins. J. Biol. Chem. 271, 15160–15165 10.1074/jbc.271.25.15160 8663016

[53] Abriel H., Rougier J. S., and Jalife J. (2015) Ion channel macromolecular complexes in cardiomyocytes: roles in sudden cardiac death. Circ. Res. 116, 1971–1988 10.1161/CIRCRESAHA.116.305017 26044251 PMC4471480

[54] Li L., Pan Z. F., Huang X., Wu B. W., Li T., Kang M. X., Ge R. S., Hu X. Y., Zhang Y. H., Ge L. J., Zhu D. Y., Wu Y. L., and Lou Y. J. (2016) Junctophilin 3 expresses in pancreatic beta cells and is required for glucose-stimulated insulin secretion. Cell Death Dis. 7, e2275 10.1038/cddis.2016.179 27336719 PMC5143404

[55] Veluthakal R., Chvyrkova I., Tannous M., McDonald P., Amin R., Hadden T., Thurmond D. C., Quon M. J., and Kowluru A. (2005) Essential role for membrane lipid rafts in interleukin-1β-induced nitric oxide release from insulin-secreting cells: potential regulation by caveolin-1+. Diabetes 54, 2576–2585 10.2337/diabetes.54.9.2576 16123345

[56] Poburko D., Kuo K. H., Dai J., Lee C. H., and van Breemen C. (2004) Organellar junctions promote targeted Ca^2+^ signaling in smooth muscle: why two membranes are better than one. Trends Pharmacol. Sci. 25, 8–15 10.1016/j.tips.2003.10.011 14723973

[57] Caldwell J. L., Smith C. E., Taylor R. F., Kitmitto A., Eisner D. A., Dibb K. M., and Trafford A. W. (2014) Dependence of cardiac transverse tubules on the BAR domain protein amphiphysin II (BIN-1). Circ. Res. 115, 986–996 10.1161/CIRCRESAHA.116.303448 25332206 PMC4274343

[58] Guo A., Zhang X., Iyer V. R., Chen B., Zhang C., Kutschke W. J., Weiss R. M., Franzini-Armstrong C., and Song L. S. (2014) Overexpression of junctophilin-2 does not enhance baseline function but attenuates heart failure development after cardiac stress. Proc. Natl. Acad. Sci. U.S.A. 111, 12240–12245 10.1073/pnas.1412729111 25092313 PMC4143026

[59] Chen B., Guo A., Zhang C., Chen R., Zhu Y., Hong J., Kutschke W., Zimmerman K., Weiss R. M., Zingman L., Anderson M. E., Wehrens X. H., and Song L. S. (2013) Critical roles of junctophilin-2 in T-tubule and excitation-contraction coupling maturation during postnatal development. Cardiovasc. Res. 100, 54–62 10.1093/cvr/cvt180 23860812 PMC3778961

[60] Fredriksson S., Gullberg M., Jarvius J., Olsson C., Pietras K., Gústafsdóttir S. M., Ostman A., and Landegren U. (2002) Protein detection using proximity-dependent DNA ligation assays. Nat. Biotechnol. 20, 473–477 10.1038/nbt0502-473 11981560

