# Supplementary material for: A junctophilin-caveolin interaction enables efficient coupling between ryanodine receptors and BKCa channels in the Ca2+ microdomain of vascular smooth muscle
Source: J Biol Chem. 2019 Jul 15;294(35):13093–105. doi: 10.1074/jbc.RA119.008342 (PMC6721949; doi:10.1074/jbc.RA119.008342)
Supplement: Supporting Information [file supp_294_35_13093__index.html]

A junctophilin-caveolin interaction enables efficient coupling between ryanodine receptors and BKCa channels in the Ca2+ microdomain of vascular smooth muscle — Junctophilin-caveolin interaction in vascular smooth muscle — A junctophilin-caveolin interaction enables efficient coupling between ryanodine receptors and BKCa channels in the Ca2+ microdomain of vascular smooth muscle — Junctophilin-caveolin interaction in vascular smooth muscle — Supporting Information 

# A junctophilin-caveolin interaction enables efficient coupling between ryanodine receptors and BKCa channels in the Ca2+ microdomain of vascular smooth muscle

## Supporting Information

- Supporting Information (to be published online) - Supporting Information to be published (Table S1-2, Fig. S1-S8, and supplementary movie legends).
- Supporting Information (to be published online) - Raw data of Fig. 5J and Table S3.
- Supplementary Movie S1 - Supplementary Movie S1
- Supplementary Movie S2 - Supplementary Movie S2
