# Supplementary material for: A junctophilin-caveolin interaction enables efficient coupling between ryanodine receptors and BKCa channels in the Ca2+ microdomain of vascular smooth muscle
Source: J Biol Chem. 2019 Jul 15;294(35):13093–105. doi: 10.1074/jbc.RA119.008342 (PMC6721949; doi:10.1074/jbc.RA119.008342)
Supplement: Supporting Information [file supp_RA119.008342_144178_2_supp_360354_pddhv1.docx]

**Supporting Information**

**A junctophilin-caveolin interaction enables efficient coupling between ryanodine receptors**

**and BK_Ca_ channels in the Ca^2+^ microdomain of vascular smooth muscle**

**Takanori Saeki, Yoshiaki Suzuki, Hisao Yamamura, Hiroshi Takeshima, and Yuji Imaizumi***

**Supplementary Figures**

- Table S1. Analysis of particle number in TIRF fields.
- Table S2. The accumulation of Ca^2+^ spark-generating sites in the signals of JP2 or Cav1.
- Table S3. The analysis about effects of Cav1 on FRET between JP2 and BK_Ca_.
- Figure S1. Expression patterns of JP1, JP2, and Cav isoforms in mMASMCs.
- Figure S2. JP2 interacts with BK_Ca_ channels in MASMCs.
- Figure S3. Localization of JP2 and BK_Ca_ channels in mMASMCs after a treatment with MβCD.
- Figure S4. BK_Ca_ channel currents in mMASMCs treated with siRNA.
- Figure S5. TIRF imaging of Ca^2+^ sparks in mMASMCs treated with siJP2.
- Figure S6. Influence of siJP2 on KCl-induced vasoconstriction and the maximal passive diameter in mesenteric artery tissues.
- Figure S7. Supposed schematic diagram of the molecular complex in the Ca^2+^ microdomain of mesenteric artery SMCs.
- Figure S8. Source data, Western blots.
- Captions for Supplementary Movie

**Supplementary Movies**

- Movie S1. Ca^2+^ images of mMASMC transiently transfected with mCherry-JP2 cDNA.
- Movie S2. Ca^2+^ images of mMASMCs transiently transfected with mCherry-Cav1 cDNA.

**Table S1. Analysis of particle number in TIRF fields.**

The number of fluorescent particles labeled with specific antibodies were counted using TIRF microscope. These particles are derived from each protein (JP2, RyR, BKα and Cav1) localized in plasma membrane or just beneath the membrane (i.e. TIRF fields approximately 200 nm from the glass bottom). In each case, treatment with MβCD (Fig, 3), deletion of Cav1 gene (Fig. 5) and knockdown of JP2 (Fig. 6) did not change the number of fluorescent particles of JP2, RyR, BKα and Cav1 within TIRF fields. Thus, these treatments are thought not to change protein levels of these protein.

|  | Control | Treated |
| --- | --- | --- |
| Fig. 3A-D. The number of RyR and JP2 before and after MβCD treatment | Control:  RyR: 38.3±9.9 (n=8)  JP2: 24.9±6.1 (n=8) | MβCD:  RyR: 41.7±9.2 (n=9), p>0.05 vs. Control  JP2: 27.6±9.0 (n=9), p>0.05 vs. Control |
| Fig. 5A-H. The number of BKα and JP2 between WT and Cav1^-/-^ | WT:  BKα: 31.3±12.1 (n=8)  JP2: 38.3±12.7 (n=8) | Cav1^-/-^:  BKα: 34.7±7.2 (n=11), p>0.05 vs. WT  JP2: 49.7±13.8 (n=11), p>0.05 vs. WT |
| Fig. 6E-H. The number of Cav1 and RyR between siControl and siJP2 | siControl:  Cav1: 51.6±11.0 (n=9)  RyR: 51.6±11.4 (n=9) | siJP2:  Cav1: 42.5±12.4 (n=8), p>0.05 vs. siControl  RyR: 45.8±15.4 (n=8), p>0.05 vs. siControl |

**Table S2. The accumulation of Ca^2+^ spark-generating sites in the mCherry signals of JP2 or Cav1.** The density of Ca^2+^ spark-generating sites in the mCherry fluorescent signals of JP2 (JP2in) or Cav1 (Cav1in) was compared with those in the outside of the signals (JP2out or Cav1out). Ca^2+^ sparks were recorded in MASMCs, in which JP2 or Cav1 labeled with mCherry was expressed as shown in Fig. 4 in the text. The area occupied by the fluorescent signals of JP2 or Cav1 in the TIRF image area in TIRF image of PM were counted (A) and the ratio (%) of sum of area occupied by JP2 or Cav1 fluorescent signals versus the total area of the TRF image of PM were calculated (B). The number of Ca^2+^ spark-generating sites in JP2 or Cav1 signals in each TIRF images were also counted. Then, the density of Ca^2+^ spark-generating sites in JP2 or Cav1 signals was compared with those outside the signals. Note that the Ca^2+^ spark-generating sites quite preferentially accumulated in JP2 and Cav1 signals.

|  | JP2in | JP2out | Cav1in | Cav1out |
| --- | --- | --- | --- | --- |
| (A) Area in TIRF images of PM (μm^2^) | 7.92±3.87 ^**^  (n=12) | 169.58±80.96 (n=12) | 3.90±2.49 ^##^ (n=15) | 153.80±79.32 (n=15) |
| (B) Area occupied in TIRF images of PM (%) | 4.55±1.76 ^**^ | 95.45±1.76 | 2.39±0.88 ^##^ | 97.61±0.88 |
| (C) Number of Ca^2+^ spark- generating sites per TIRF image (sites/image) | 1.83±1.11 | 1.17±1.03 | 1.60±1.18 ^#^ | 2.87±1.36 |
| (D) Density of Ca^2+^ spark- generating sites (sites/μm^2^) | 0.27±0.26 ^**^ | 0.007±0.007 | 0.52±0.45 ^##^ | 0.020±0.010 |

TIRF: Total Internal Reflection Microscopy

PM: plasma membrane

Number in parenthesis indicates the number of cells examined.

**: p < 0.01 vs. JP2out

#: p < 0.05 and ##: p < 0.01 vs. Cav1out

**Table S3: The analysis about effects of Cav1 on FRET between JP2 and BK_Ca_.** 2-Way ANOVA was applied to analyze the data shown in Fig. 5J. The Main Effect 1 includes the effect of Cav1 compared in WT and Cav^-/-^ on the FRET values measured as the molecular interaction between YFP-JP2 and BKα-CFP. The Main Effect 2 was set to evaluate the molecular interaction between BKα-CFP and YFP alone, as the control, in comparison with that between YFP-JP2 and BKα-CFP. The Interaction between Main Effect 1 and Main Effect 2 is statistically significant (F-test, p <0.05). Thus, the statistical significance between the four groups was examined by Tukey’s test in Fig. 5J.

| The 2-way ANOVA | SS (sum of squares) | DF (degrees of freedom) | MS (Mean square) | F ratio | 5% F ratio | p value |
| --- | --- | --- | --- | --- | --- | --- |
| Main Effect 1 (WT, Cav1^-/-^) | 1122.18 | 1 | 1122.18 | 14.11 | 3.90 | 0.000236 |
| Main Effect 2 (YFP-JP2, YFP) | 4026.53 | 1 | 4026.53 | 50.62 | 3.90 | 2.85E-11 |
| Interaction | 615.60 | 1 | 615.60 | 7.74 | 3.90 | 0.006002 |
| Error (within) | 13760.44 | 173 | 79.54 |  |  |  |
| Total | 19524.75 | 176 |  |  |  |  |


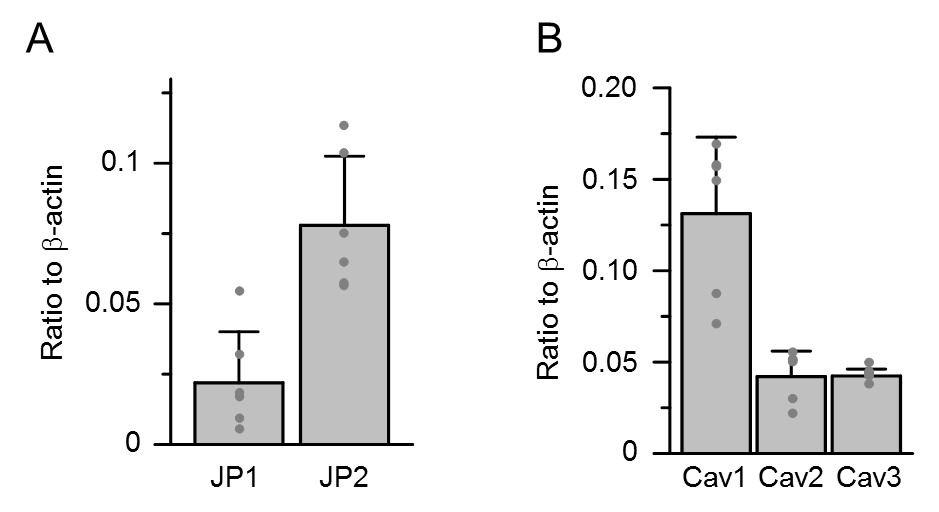


**Figure S1. Expression patterns of JP1, JP2, and Cav isoforms in mMASMCs.** The mRNA expression of JP1 and 2 (**A**) and Cav1, 2, and 3 (**B**) in mMASMCs is profiled based on quantitative real-time PCR analyses. mRNA expression levels were normalized to endogenous β-actin (JP1: 0.022 ± 0.018, JP2: 0.078 ± 0.025, Cav1: 0.131 ± 0.042, Cav2: 0.042 ± 0.014, Cav3: 0.042 ± 0.004, n=6).


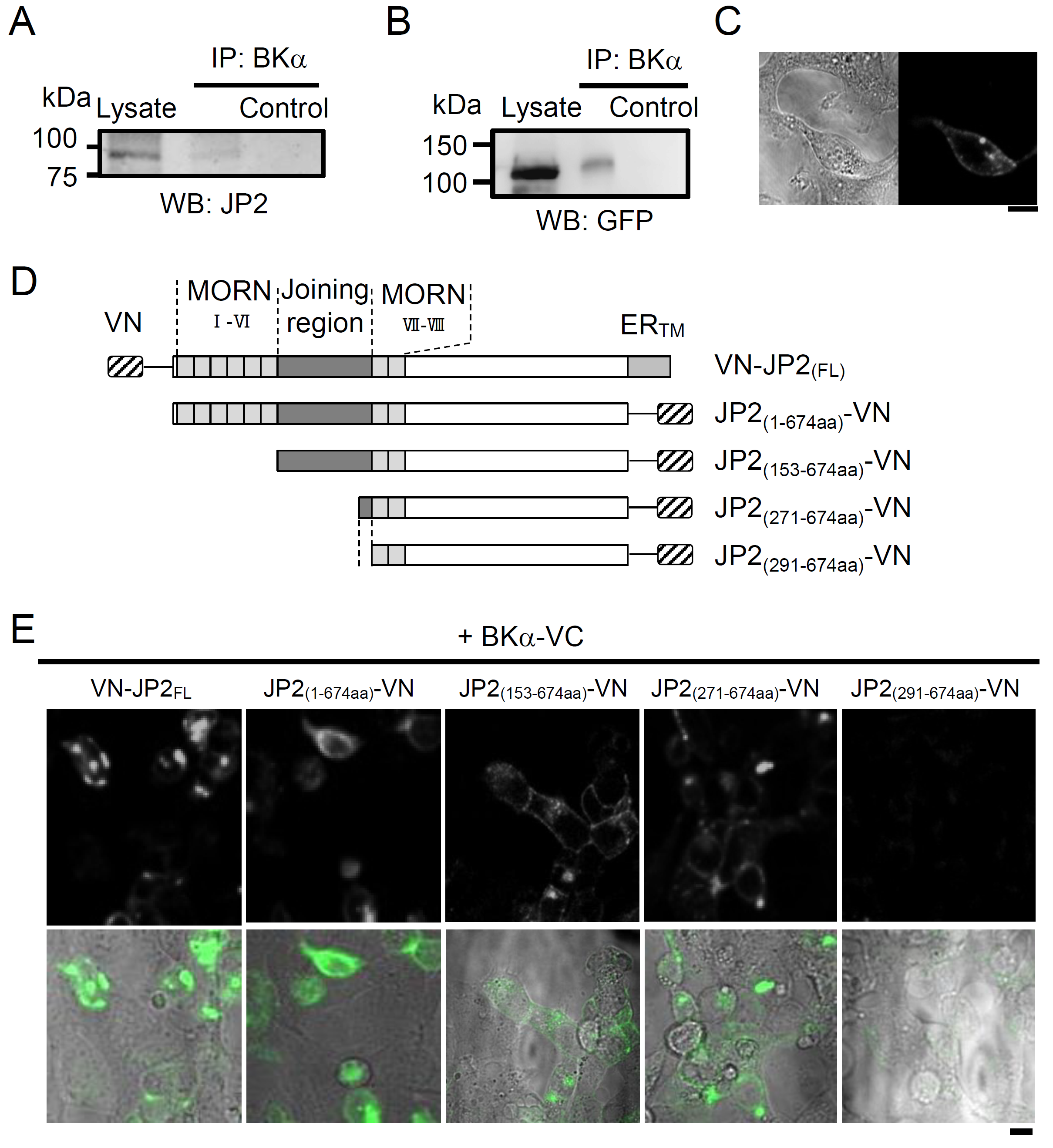


**Fig. S2. JP2 interacts with BK_Ca_ channels in MASMCs.** (**A, B**) Co-IP was performed to detect molecular coupling between JP2 and BK_Ca_ channel α subunits in the rat mesenteric artery (A) and HEK293 cells co-expressing nGFP-JP2 and BKα-mCherry (**B**). Extracts were processed for IP with anti-BKα antibodies. (**C**) A representative image of the BiFC assay. Fluorescent signals indicate the reconstruction of Venus by the binding of VN-JP2 and BKα-VC. (**D**) The JP2 truncation constructs used in the present study are shown. MORN motifs, the joining region, and ER transmembrane domain (ER_TM_) are also indicated. All VN-tagged JP2 constructs were co-expressed with BKα (BKα-VC) in HEK293 cells. (**E**) Series of representative confocal images from the BiFC assay. Fluorescent signals indicate the complementation of VN and VC. Note that JP2_(291-674aa)_ showed no fluorescence, while JP2_(271-674aa)_ showed clear Venus fluorescence. These results indicate that 271-290 residues in the JP2 joining region are the essential site for binding with BK_Ca_ channels. Scale bars denote 10 μm.

**
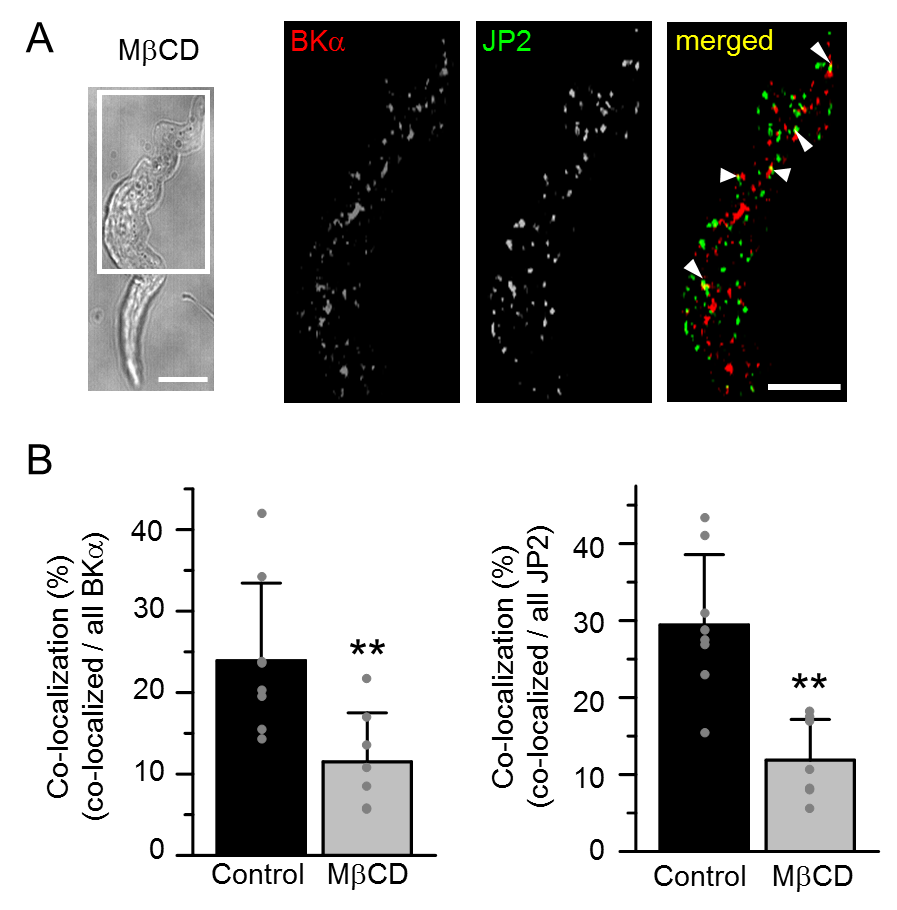
**

**Figure S3.** **Localization of JP2 and BK_Ca_ channels in mMASMCs after a treatment with MβCD.** (**A**) JP2 and BKα proteins in freshly isolated mMASMCs from WT mice were labeled with specific antibodies after a treatment with MβCD and then visualized under a TIRF microscope. The areas of myocytes denoted by white square lines in transmitted light images were shown in fluorescent images. Fluorescent signals corresponding to JP2, BKα, and their co-localization are shown in green, red, and yellow (indicated by arrowheads), respectively. Scale bars denote 10 μm. (**B**) The ratio of the number of co-localized particles to that of total BK_Ca_ channels (11.9±5.3%, red column) or JP2 (11.4±6.0%, green column) particles in control (n=8) and MβCD myocytes (n=7). Data of the control are the same as those of WT in Fig. 4C and D, **p<0.01; the Student’s *t*-test. All data are expressed as the mean ± s.d.


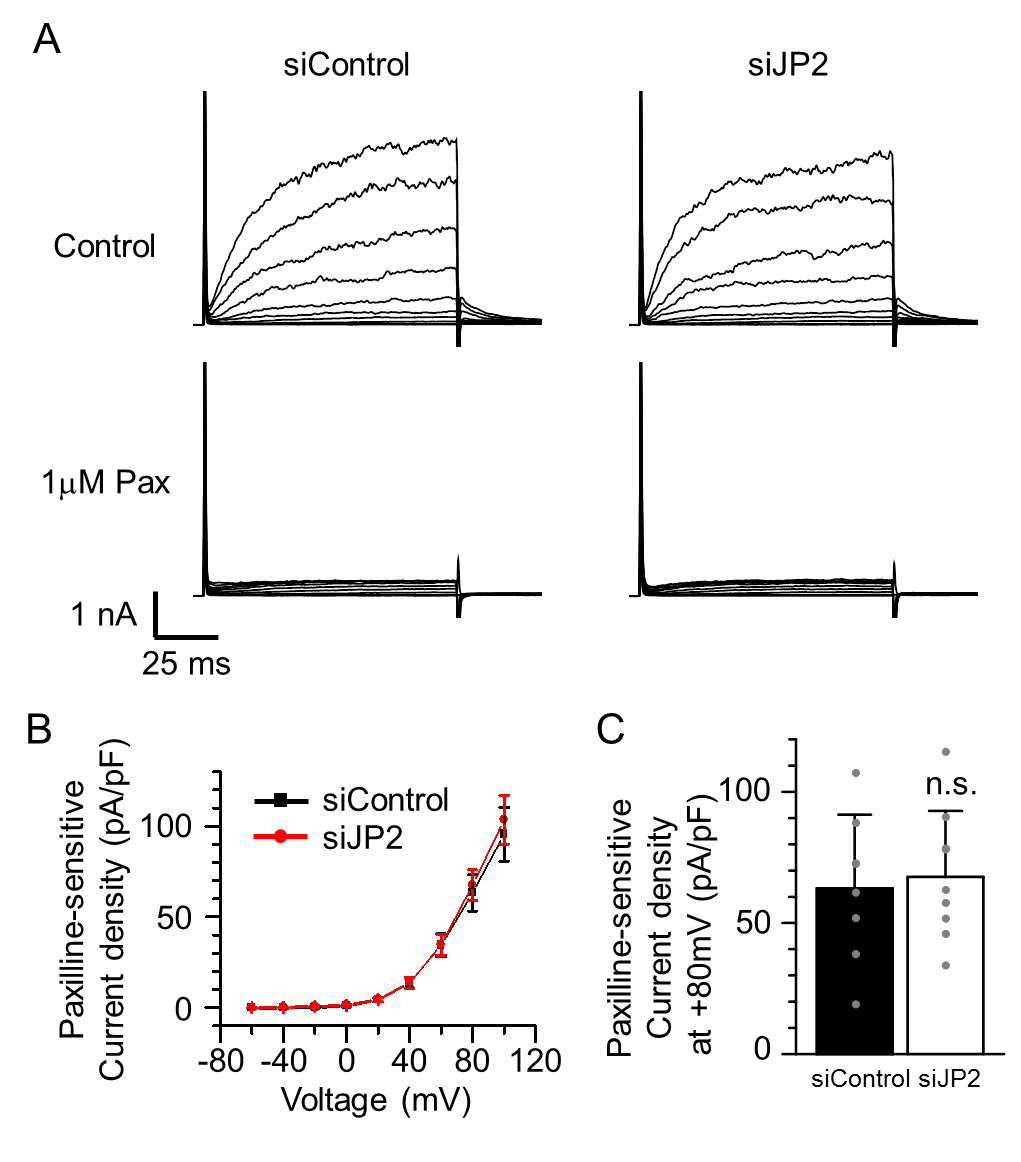


**Figure S4. BK_Ca_ channel currents in mMASMCs treated with the siRNA of JP2.** (**A**) Representative BK_Ca_ channel currents in cells pre-treated with siControl or siJP2 were recorded under the whole-cell voltage-clamp mode. These BK_Ca_ currents were activated from a holding potential of −80 mV by applying 100-ms voltage clamp steps between −60 and 80 mV in increments of 20 mV at a rate of 0.1 Hz. The BK_Ca_ current was identified as 1 μM paxilline (Pax)-sensitive currents. (**B**) Current density-voltage relationships of 1 μM Pax-sensitive currents are summarized. (**C**) The current density of 1 μM Pax-sensitive currents at +80 mV was compared between two groups (siControl: n=6, siJP2: n=10). p>0.05; the Student’s *t*-test. All data are expressed as the mean ± s.d. The numbers of cells are shown in parentheses.


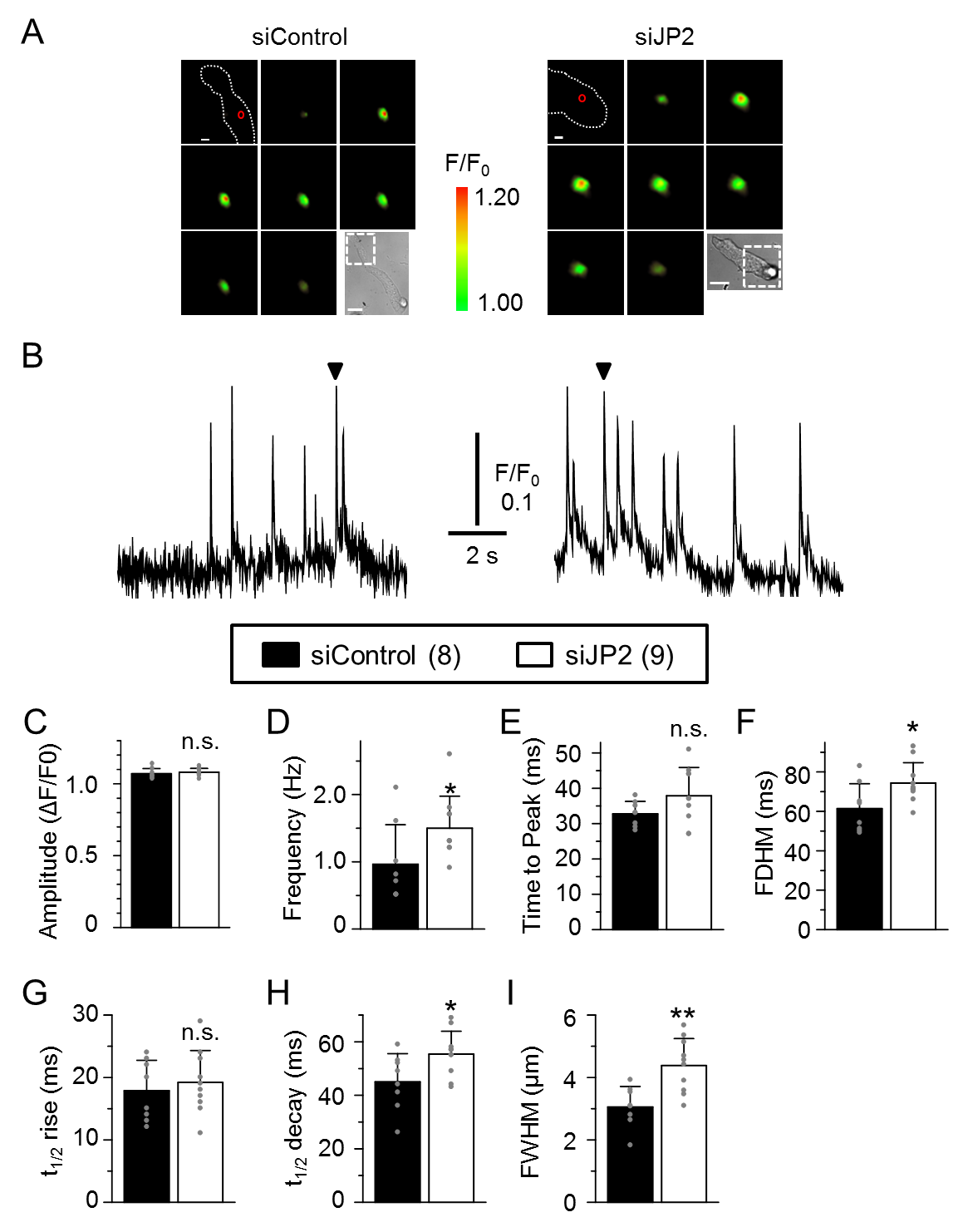


**Figure S5. TIRF imaging of Ca^2+^ sparks in mMASMCs treated with siJP2.** (**A**) Representative images of [Ca^2+^]_i_ changes in mMASMCs treated with siControl or siJP2 were collected at the time points indicated by arrowheads in (**B**). The scale bars in transfer and fluorescent images indicate 10 and 2 μm, respectively. The areas of myocytes denoted by white broken lines in transmitted light images were shown in fluorescent images. Fluorescent images were obtained at 70 Hz using a TIRF microscope. (**B**) Recordings of changes in [Ca^2+^]_i_ at the sites circled in the fluorescent images (**A**) are shown in a longer time-course. *F* denotes the average fluorescent intensity within the regions of interests (1 μm in diameter). *F_0_* is the basal fluorescence intensity of the ROIs. (**C, D**) Summary of the mean Ca^2+^ spark amplitude (**C**), frequency (**D**), time to peak (**E**), full duration at half-maximum (FDHM) (**F**), t_1/2_ rise (**G**), t_1/2_ decay (**H**), and full-width half-maximum amplitude (FWHM) (**I**) in myocytes treated with siControl (n=8) or siJP2 (n=10). *p<0.05, **p<0.01; the Student’s *t*-test. All data are expressed as the mean ± s.d. The numbers of cells examined are shown in parentheses.


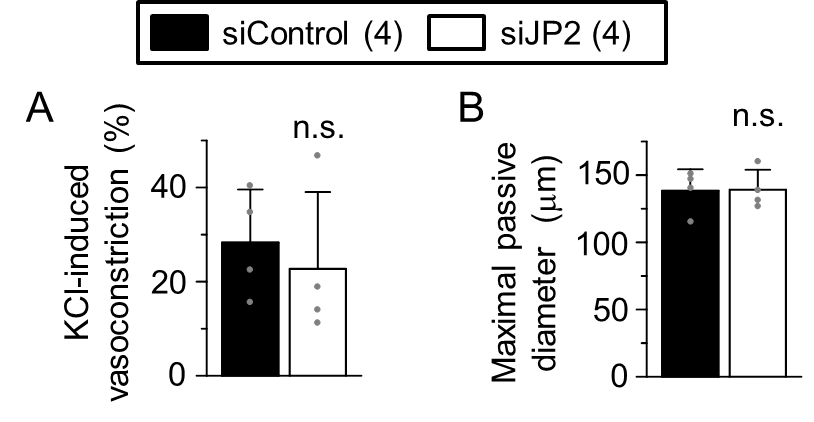


**Figure S6. Influence of siJP2 on KCl-induced vasoconstriction and the maximal passive diameter in mesenteric artery tissues. (A)** Summary of the constriction in siRNA-treated mesenteric artery segments measured by pressure myography in response to 40 mM extracellular [K^+^] (n=4). **(B)** Summary of the maximal passive diameter of mesenteric artery segments treated with siRNA. The maximal relaxation was induced by bathing in Ca^2+^-free Kreb’s solution (siControl: 138.4±16.0 μm, siJP2: 139.2±14.8 μm, n=4). P>0.05; Student’s *t*-test. All data are expressed as the mean ± s.d.


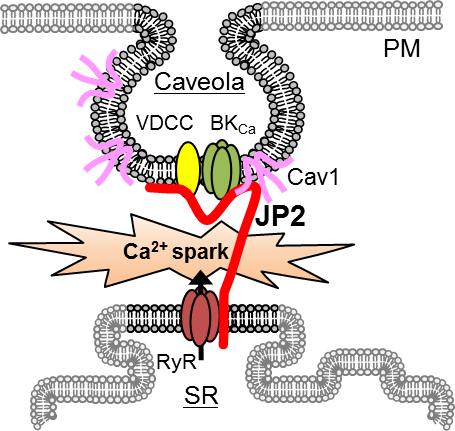


**Figure S7. Supposed schematic diagram of the molecular complex in the Ca^2+^ microdomain of mesenteric artery SMCs.** Note the key role of the Cav1-JP2 interaction in the formation of the Ca^2+^ microdomain.


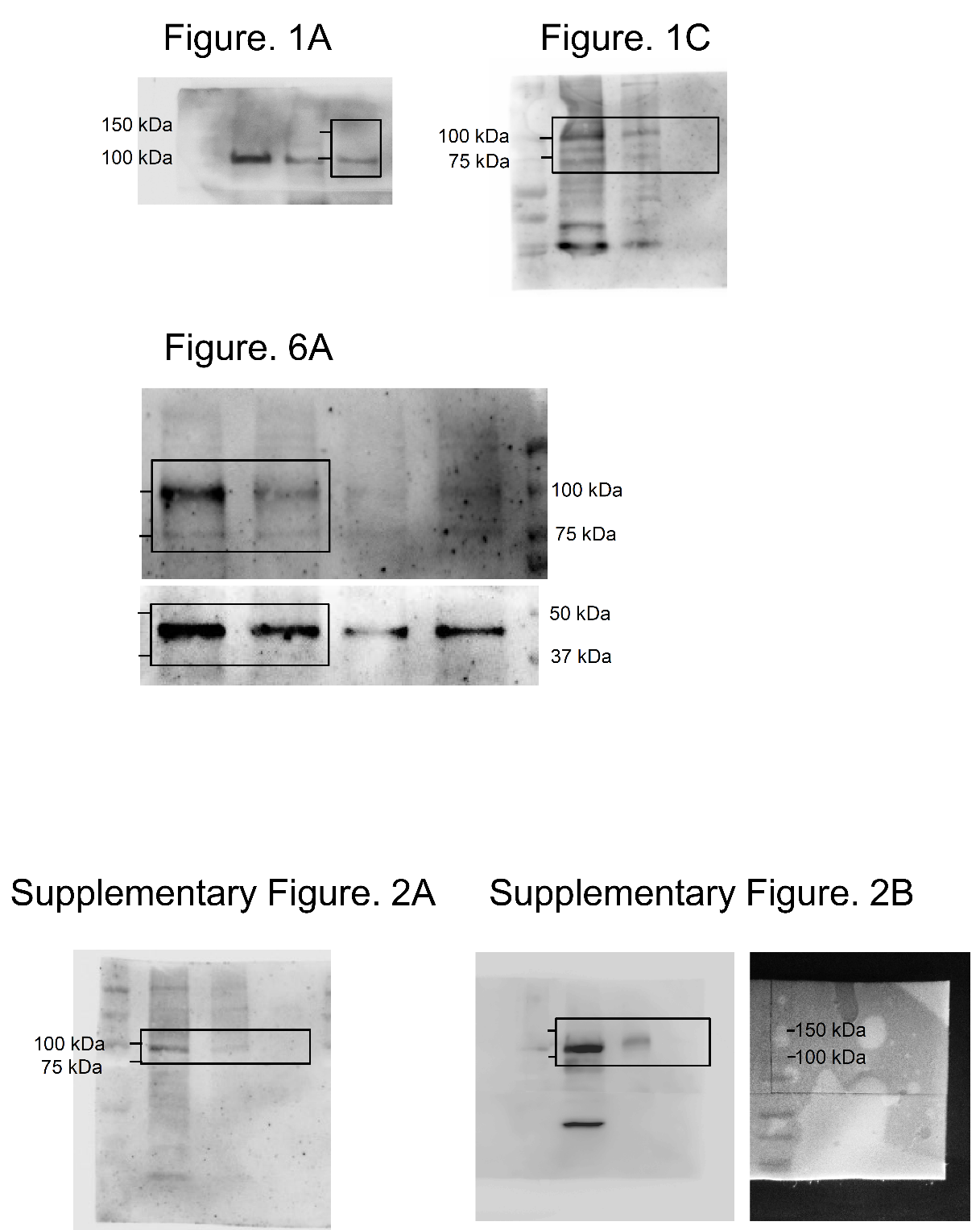


**Figure S8. Source data, Western blots.**

**Captions for Supplementary Movie**

**Movie S1. Ca^2+^ images of mMASMC transiently transfected with mCherry-JP2 cDNA.** Single molecules or clusters of mCherry-JP2 are shown as purple dots. Changes in [Ca^2+^]_i_ are simultaneously recorded using fluo4/AM. The field of view is the same as that shown in Fig. 2I. This movie and all others in this supplement run at a reduced by (1/2) time-scale.

**Movie S2. Ca^2+^ images of mMASMCs transiently transfected with mCherry-Cav1 cDNA.** Single molecules or clusters of mCherry-Cav1 are shown as purple dots. Changes in [Ca^2+^]_i_ are simultaneously recorded using fluo4/AM. The field of view is the same as that shown in Fig. 2L.
